# Global estimates on the number of people blind or visually impaired by Uncorrected Refractive Error: a meta-analysis from 2000 to 2020

**DOI:** 10.1038/s41433-024-03106-0

**Published:** 2024-07-04

**Authors:** Vision Loss Expert Group of the Global Burden of Disease Study, Julie-Anne Little, Nathan G. Congdon, Serge Resnikoff, Tasanee Braithwaite, Janet Leasher, Kovin Naidoo, Tim Fricke, Ian Tapply, Arthur G. Fernandes, Maria Vittoria Cicinelli, Alessandro Arrigo, Nicolas Leveziel, Hugh R. Taylor, Tabassom Sedighi, Seth Flaxman, Maurizio Battaglia Parodi, Mukkharram M. Bikbov, Alain Bron, Ching-Yu Cheng, Monte A. Del Monte, Joshua R. Ehrlich, Leon B. Ellwein, David Friedman, João M. Furtado, Gus Gazzard, Ronnie George, M. Elizabeth Hartnett, Jost B. Jonas, Rim Kahloun, John H. Kempen, Moncef Khairallah, Rohit C. Khanna, Judy E. Kim, Van Charles Lansingh, Vinay Nangia, Michal Nowak, Konrad Pesudovs, Tunde Peto, Pradeep Ramulu, Fotis Topouzis, Mitiadis Tsilimbaris, Ya Xing Wang, Ningli Wang, Rupert R. A. Bourne, the GBD 2019 Blindness and Vision Impairment Collaborators, Julie-Anne Little, Nathan G. Congdon, Serge Resnikoff, Tasanee Braithwaite, Janet L. Leasher, Kovin S. Naidoo, Nina Tahhan, Timothy Fricke, Arthur G. Fernandes, Maria Vittoria Cicinelli, Alessandro Arrigo, Nicolas Leveziel, Paul Svitil Briant, Theo Vos, Seth Flaxman, Yohannes Habtegiorgis Abate, Zahra Abbasi Dolatabadi, Michael Abdelmasseh, Mohammad Abdollahi, Ayele Mamo Abebe, Olumide Abiodun, Richard Gyan Aboagye, Woldu Aberhe Abrha, Hiwa Abubaker Ali, Eman Abu-Gharbieh, Salahdein Aburuz, Tadele Girum Girum Adal, Lawan Hassan Adamu, Nicola J. Adderley, Isaac Yeboah Addo, Tayo Alex Adekiya, Kishor Adhikari, Qorinah Estiningtyas Sakilah Adnani, Saira Afzal, Shahin Aghamiri, Antonella Agodi, Williams Agyemang-Duah, Bright Opoku Ahinkorah, Aqeel Ahmad, Hooman Ahmadzadeh, Ayman Ahmed, Haroon Ahmed, Fares Alahdab, Mohammed Albashtawy, Mohammad T. AlBataineh, Tsegaye Alemu, Ahmad Samir Alfaar, Fadwa Alhalaiqa Naji Alhalaiqa, Robert Kaba Alhassan, Abid Ali, Syed Shujait Shujait Ali, Louay Almidani, Karem H. Alzoubi, Sofia Androudi, Rodrigo Anguita, Abhishek Anil, Anayochukwu Edward Anyasodor, Jalal Arabloo, Aleksandr Y. Aravkin, Damelash Areda, Akeza Awealom Asgedom, Mubarek Yesse Ashemo, Tahira Ashraf, Seyyed Shamsadin Athari, Bantalem Tilaye Tilaye Atinafu, Maha Moh’d Wahbi Atout, Alok Atreya, Haleh Ayatollahi, Ahmed Y. Azzam, Sara Bagherieh, Ruhai Bai, Atif Amin Baig, Freddie Bailey, Ovidiu Constantin Baltatu, Shirin Barati, Martina Barchitta, Mainak Bardhan, Till Winfried Bärnighausen, Amadou Barrow, Maurizio Battaglia Parodi, Nebiyou Simegnew Bayileyegn, Alemshet Yirga Berhie, Abhishek Bhadra, Akshaya Srikanth Srikanth Bhagavathula, Pankaj Bhardwaj, Sonu Bhaskar, Ajay Nagesh Bhat, Gurjit Kaur Bhatti, Mukharram Bikbov, Marina G. Birck, Yasser Bustanji, Zahid A. Butt, Florentino Luciano Caetano dos Santos, Vera L. A. Carneiro, Muthia Cenderadewi, Gashaw Sisay Chanie, Nicolas Cherbuin, Dinh-Toi Chu, Kaleb Coberly, Natália Cruz-Martins, Omid Dadras, Xiaochen Dai, Lalit Dandona, Rakhi Dandona, Ana Maria Dascalu, Anna Dastiridou, Tadesse Asmamaw Dejenie, Dessalegn Demeke, Diriba Dereje, Nikolaos Dervenis, Vinoth Gnana Chellaiyan Devanbu, Daniel Diaz, Mengistie Diress, Thanh Chi Do, Thao Huynh Phuong Do, Arkadiusz Marian Dziedzic, Hisham Atan Edinur, Joshua R. Ehrlich, Michael Ekholuenetale, Hala Rashad Elhabashy, Muhammed Elhadi, Mohammad Hassan Emamian, Mehdi Emamverdi, Azin Etemadimanesh, Adeniyi Francis Fagbamigbe, Hossein Farrokhpour, Ali Fatehizadeh, Alireza Feizkhah, Lorenzo Ferro Desideri, Getahun Fetensa, Florian Fischer, Ali Forouhari, João M. Furtado, Muktar A. Gadanya, Abhay Motiramji Gaidhane, Aravind P. Gandhi, Tilaye Gebru Gebi, Mesfin Gebrehiwot, Gebreamlak Gebremedhn Gebremeskel, Yibeltal Yismaw Gela, Bardiya Ghaderi Yazdi, Khalil Ghasemi Falavarjani, Fariba Ghassemi, Sherief Ghozy, Ali Golchin, Mahaveer Golechha, Pouya Goleij, Shi-Yang Guan, Sapna Gupta, Vivek Kumar Gupta, Rasool Haddadi, Teklehaimanot Gereziher Haile, Billy Randall Hammond, Mehdi Harorani, Ahmed I. Hasaballah, Ikramul Hasan, Hamidreza Hasani, Hossein Hassanian-Moghaddam, Golnaz Heidari, Demisu Zenbaba Heyi, Ramesh Holla, Mehdi Hosseinzadeh, Chengxi Hu, Hong-Han Huynh, Bing-Fang Hwang, Ivo Iavicoli, Irena M. Ilic, Mustapha Immurana, Sheikh Mohammed Shariful Islam, Louis Jacob, Abdollah Jafarzadeh, Mihajlo Jakovljevic, Manthan Dilipkumar Janodia, Sathish Kumar Jayapal, Shubha Jayaram, Jost B. Jonas, Nitin Joseph, Charity Ehimwenma Joshua, Sagarika Kamath, Himal Kandel, Ibraheem M. Karaye, Hengameh Kasraei, Soujanya Kaup, Harkiran Kaur, Navjot Kaur, Gbenga A. Kayode, John H. Kempen, Yousef Saleh Khader, Himanshu Khajuria, Rovshan Khalilov, Ajmal Khan, Moawiah Mohammad Khatatbeh, Mahalaqua Nazli Khatib, Biruk Getahun Kibret, Yun Jin Kim, Adnan Kisa, Sezer Kisa, Soewarta Kosen, Ai Koyanagi, Kewal Krishan, Burcu Kucuk Bicer, Nithin Kumar, L. V. Simhachalam Kutikuppala, Chandrakant Lahariya, Tri Laksono, Dharmesh Kumar Lal, Van Charles Lansingh, Munjae Lee, Seung Won Lee, Wei-Chen Lee, Stephen S. Lim, Xuefeng Liu, Sandeep B. Maharaj, Alireza Mahmoudi, Kashish Malhotra, Ahmad Azam Malik, Iram Malik, Tauqeer Hussain Mallhi, Vahid Mansouri, Roy Rillera Marzo, Andrea Maugeri, Gebrekiros Gebremichael Meles, Abera M. Mersha, Tomislav Mestrovic, Ted R. Miller, Mehdi Mirzaei, Awoke Misganaw, Sanjeev Misra, Prasanna Mithra, Soheil Mohammadi, Abdollah Mohammadian-Hafshejani, Maryam Mohammadzadeh, Hoda Mojiri-forushani, Ali H. Mokdad, Hamed Momeni-Moghaddam, Fateme Montazeri, Maryam Moradi, Parsa Mousavi, Christopher J. L. Murray, Ganesh R. Naik, Gurudatta Naik, Zuhair S. Natto, Muhammad Naveed, Biswa Prakash Nayak, Hadush Negash, Seyed Aria Nejadghaderi, Dang H. Nguyen, Duc Hoang Nguyen, Hien Quang Nguyen, Phat Tuan Nguyen, Van Thanh Nguyen, Robina Khan Niazi, Efaq Ali Noman, Bogdan Oancea, Osaretin Christabel Okonji, Andrew T. Olagunju, Isaac Iyinoluwa Olufadewa, Obinna E. Onwujekwe, Abdulahi Opejin Opejin, Michal Ordak, Uchechukwu Levi Osuagwu, Nikita Otstavnov, Mayowa O. Owolabi, Jagadish Rao Padubidri, Songhomitra Panda-Jonas, Anamika Pandey, Shahina Pardhan, Amirhossein Parsaei, Jay Patel, Shrikant Pawar, Arokiasamy Perianayagam, Navaraj Perumalsamy, Konrad Pesudovs, Ionela-Roxana Petcu, Hoang Tran Pham, Mohsen Pourazizi, Elton Junio Sady Prates, Ibrahim Qattea, Pankaja Raghav Raghav, Mohammad Hifz Ur Rahman, Mosiur Rahman, Shakthi Kumaran Ramasamy, Premkumar Ramasubramani, Mohammad-Mahdi Rashidi, Elrashdy Moustafa Mohamed Redwan, Nazila Rezaei, Jefferson Antonio Buendia Rodriguez, Zahra Saadatian, Siamak Sabour, Basema Saddik, Umar Saeed, Sare Safi, Amene Saghazadeh, Fatemeh Saheb Sharif-Askari, Narjes Saheb Sharif-Askari, Amirhossein Sahebkar, Mohammad Ali Sahraian, Joseph W. Sakshaug, Mohamed A. Saleh, Sara Samadzadeh, Yoseph Leonardo Samodra, Abdallah M. Samy, Mete Saylan, Siddharthan Selvaraj, Yashendra Sethi, Allen Seylani, Moyad Jamal Shahwan, Masood Ali Shaikh, Muhammad Aaqib Shamim, Bereket Beyene Shashamo, Wondimeneh Shibabaw Shiferaw, Mika Shigematsu, Aminu Shittu, Parnian Shobeiri, Seyed Afshin Shorofi, Migbar Mekonnen Sibhat, Emmanuel Edwar Siddig, Juan Carlos Silva, Jasvinder A. Singh, Paramdeep Singh, Houman Sotoudeh, Raúl A. R. C. Sousa, Chandrashekhar T. Sreeramareddy, Mohammad Tabish, Majid Taheri, Yao Tan, Birhan Tsegaw Taye, Mohamad-Hani Temsah, Jansje Henny Vera Ticoalu, Tala Tillawi, Misganaw Guadie Tiruneh, Aristidis Tsatsakis, Guesh Mebrahtom Tsegay, Miltiadis K. Tsilimbaris, Sree Sudha Ty, Chukwudi S. Ubah, Muhammad Umair, Sahel Valadan Tahbaz, Rohollah Valizadeh, Maria Viskadourou, Gizachew Tadesse Wassie, Nuwan Darshana Wickramasinghe, Guadie Sharew Wondimagegn, Galal Yahya, Lin Yang, Yao Yao, Arzu Yiğit, Yazachew Yismaw, Naohiro Yonemoto, Yuyi You, Mikhail Sergeevich Zastrozhin, Getachew Assefa Zenebe, Zhi-Jiang Zhang, Hanqing Zhao, Magdalena Zielińska, Mohammad Zoladl, Jaimie D. Steinmetz, Rupert Bourne

**Affiliations:** 1 Biomedical Sciences Research Institute, Ulster University, Coleraine, BT52 1SA UK; 2 Queen’s University Belfast, Northern Ireland, UK; 3 Orbis International, New York, USA; 4 Zhongshan Ophthalmic Center, Sun Yat-sen University, Guangzhou, China; 5 Brien Holden Vision Institute, Sydney, NSW Australia; 6 School of Optometry and Vision Sciences, Faculty of Medicine, University of New South Wales, Sydney, NSW Australia; 7 School of Life Course and Population Sciences, King’s College London, London, UK; 8 The Medical Eye Unit, Guy’s and St Thomas’ NHS Foundation Trust, London, UK; 9 Nova Southeastern University College for Optometry, Fort Lauderdale, Florida USA; 10 African Vision Research Institute, University of KwaZulu-Natal (UKZN), Durban, South Africa; 11 School of Optometry and Vision Science, University of New South Wales, Sydney, Australia; 12 Australian College of Optometry, Vic, Australia; 13 University of Melbourne, Vic, Australia; 14 UNSW Sydney, Sydney, NSW Australia; 15 Department of Ophthalmology, Cambridge University Hospitals, Cambridge, UK; 16 Federal University of Sao Paolo, Sao Paolo/SP, Brazil; 17 University of Calgary, Calgary/AB, Canada; 18 School of Medicine, Vita-Salute San Raffaele University, Milan, Italy; 19 Department of Ophthalmology, IRCCS San Raffaele Scientific Institute, Milan, Italy; 20 Scientific Institute San Raffaele Hospital, Vita-Salute University, Milan, Italy; 21 University of Poitiers, Poitiers, France; 22 CHU de Poitiers, Poitiers, France; 23 School of Population and Global Health, University of Melbourne, Carlton, VIC, Australia; 24 Vision and Eye Research Institute, Anglia Ruskin University, Cambridge, UK; 25 Department of Computer Science, University of Oxford, Oxford, UK; 26 Department of Ophthalmology, Vita-Salute San Raffaele University, Milano, Italy; 27 Ufa Eye Research Institute, Ufa, Russia; 28 University Hospital, Dijon, France; 29 National University of Singapore, Singapore, Singapore; 30 Singapore Eye Research Institute, Singapore, Singapore; 31 University of Michigan, Singapore, Singapore; 32 Kellogg Eye Center, Ann Arbor, MI 48105 USA; 33 Institute for Social Research, University of Michigan, Michigan, USA; 34 Department of Ophthalmology and Visual Sciences, University of Michigan, Michigan, USA; 35 National Eye Institute, Bethesda, MD USA; 36 Mass Eye and Ear, Harvard Medical School, Boston, MA 02115 USA; 37 Ribeirão Preto Medical School, University of São Paulo, São Paulo, Brazil; 38 Institute of Ophthalmology UCL & NIHR Biomedical Research Centre, Bethesda, MD USA; 39 Sankara Nethralaya, Medical Research Foundation, Chennai, 600006 India; 40 Stanford University, Stanford, CA 94305 USA; 41 Department of Ophthalmology, Medical Faculty Mannheim, Heidelberg University, Heidelberg, Germany; 42 Associated Ophthalmologists of Monastir, Monastir, Tunisia; 43 Department of Ophthalmology, Harvard University, Boston, MA USA; 44 Eye Unit, MyungSung Medical College, Addis Ababa, Ethiopia; 45 Department of Ophthalmology, Addis Ababa University, Addis Ababa, Ethiopia; 46 Sight for Souls, Bellevue, WA USA; 47 Fattouma Bourguiba University Hospital, University of Monastir, Monastir, 5000 Tunisia; 48 Allen Foster Community Eye Health Research Centre, Gullapalli Pratibha Rao International Centre for Advancement of Rural Eye care, L.V. Prasad Eye Institute, Hyderabad, India; 49 Brien Holden Eye Research Centre, L.V. Prasad Eye Institute, Banjara Hills, Hyderabad, India; 50 University of Rochester, School of Medicine and Dentistry, Rochester, NY USA; 51 University of Texas Southwestern Medical Center, Dallas, TX 75390 USA; 52 HelpMeSee, Instituto Mexicano de Oftalmologia, New York, NY 10018-8005 USA; 53 University of Miami, Coral Gables, FL 33146 USA; 54 University of Utah, Salt Lake City, UT 84112 USA; 55 Suraj Eye Instate, 559, New colony, Nagpur, India; 56 Institute of Optics and Optometry, University of Social Science, 121 Gdanska str., Lodz, 90-519 Poland; 57 Medicine & Health, University of New South Wales, Sydney, NSW Australia; 58 Centre for Public Health, Queens University Belfast, Northern Ireland, Belfast, BT15 1ED UK; 59 John Hopkins Wilmer Eye Institute, Baltimore, USA; 60 1st Department of Ophthamology, Medical School, Aristotle University of Thessaloniki, Ahepa Hospital, Thessaloniki, 546 36 Greece; 61 University of Crete Medical School, Giofirakia, 715 00 Greece; 62 Beijing Institute of Ophthamology, Beijing Tongren Hospital, Capital Medical University, Beijing Ophthamology and Visual Sciences Key Laboratory, Beijing, China; 63 Beijing Institute of Ophthamology, Beijing Tongren Eye Center, Beijing Tongren Hospital, Capital Medical University, Beijing, China; 64 School of Biomedical Sciences, Ulster University, Coleraine, UK; 65 Centre for Public Health, Queen’s University, Belfast, UK; 66 ORBIS International, New York, NY USA; 67 School of Optometry and Vision Science, University of New South Wales, Sydney, NSW Australia; 68 Ophthalmology Department, Moorfields Eye Hospital NHS Foundation Trust, London, UK; 69 International Centre for Eye Health, London School of Hygiene & Tropical Medicine, London, UK; 70 College of Optometry, Nova Southeastern University, Fort Lauderdale, FL USA; 71 Discipline of Optometry, University of KwaZulu-Natal, Durban, South Africa; 72 Department of Optometry and Vision Sciences, University of Melbourne, Melbourne, VIC Australia; 73 Department of Ophthalmology and Visual Sciences, Federal University of São Paulo, Sao Paulo, Brazil; 74 Department of Ophthalmology, San Raffaele Scientific Institute, Milano, Italy; 75 Scientific Institute San Raffaele Hospital, Vita-Salute San Raffaele University, Milan, Italy; 76 Ophthalmology Department, CHU de Poitiers (Poitiers University Hospital), Poitiers, France; 77 Unité 1084, National Institute of Health and Medical Research (INSERM), Poitiers, France; 78 Institute for Health Metrics and Evaluation, University of Washington, Seattle, WA USA; 79 Department of Health Metrics Sciences, School of Medicine, University of Washington, Seattle, WA USA; 80 Department of Mathematics, Imperial College London, London, UK; 81 Department of Clinical Governance and Quality Improvement, Aleta Wondo Hospital, Aleta Wondo, Ethiopia; 82 Department of Medical-Surgical Nursing, Tehran University of Medical Sciences, Tehran, Iran; 83 Department of Surgery, Marshall University, Huntington, WV USA; 84 The Institute of Pharmaceutical Sciences (TIPS), Tehran University of Medical Sciences, Tehran, Iran; 85 School of Pharmacy, Tehran University of Medical Sciences, Tehran, Iran; 86 Pediatrics Nursing Department, Debre Berhan University, Debre Berhan, Ethiopia; 87 Department of Community Medicine, Babcock University, Ilishan-Remo, Nigeria; 88 Department of Family and Community Health, University of Health and Allied Sciences, Ho, Ghana; 89 Department of Adult Health Nursing, Aksum University, Aksum, Ethiopia; 90 Department of Banking and Finance, University of Human Development, Sulaymaniyah, Iraq; 91 Clinical Sciences Department, University of Sharjah, Sharjah, United Arab Emirates; 92 Department of Therapeutics, United Arab Emirates University, Al Ain, United Arab Emirates; 93 College of Pharmacy, University of Jordan, Amman, Jordan; 94 Department of Public Health, Wolkite University, Wolkite, Ethiopia; 95 Department of Human Anatomy, Federal University Dutse, Dutse, Nigeria; 96 Institute of Applied Health Research, University of Birmingham, Birmingham, UK; 97 Centre for Social Research in Health, University of New South Wales, Sydney, NSW Australia; 98 Quality and Systems Performance Unit, Cancer Institute NSW, Sydney, NSW Australia; 99 Department of Pharmaceutical Sciences, Howard University, Washington, DC USA; 100 School of Public Health, Chitwan Medical College and Teaching Hospital, Bharatpur, Nepal; 101 Public Health Section, Himalayan Environment and Public Health Network (HEPHN), Chitwan, Nepal; 102 Faculty of Medicine, Padjadjaran University, Bandung, Indonesia; 103 Department of Community Medicine, King Edward Memorial Hospital, Lahore, Pakistan; 104 Department of Public Health, Public Health Institute, Lahore, Pakistan; 105 Department of Biotechnology, Shahid Beheshti University of Medical Sciences, Tehran, Iran; 106 Department of Medical and Surgical Sciences and Advanced Technologies “G.F. Ingrassia”, University of Catania, Catania, Italy; 107 Department of Geography and Planning, Queen’s University, Kingston, ON Canada; 108 School of Public Health, University of Technology Sydney, Sydney, NSW Australia; 109 Department of Medical Biochemistry, Shaqra University, Shaqra, Saudi Arabia; 110 Bascom Palmer Eye Institute, University of Miami, Miami, FL USA; 111 Institute of Endemic Diseases, University of Khartoum, Khartoum, Sudan; 112 Swiss Tropical and Public Health Institute, University of Basel, Basel, Switzerland; 113 Department of Biosciences, COMSATS Institute of Information Technology, Islamabad, Pakistan; 114 Evidence-Based Practice Center, Mayo Clinic Foundation for Medical Education and Research, Rochester, MN USA; 115 Community and Mental Health Department, Al al-Bayt University, Mafraq, Jordan; 116 Department of Molecular Biology and Genetics, Khalifa University, Abu Dhabi, United Arab Emirates; 117 Department of Public Health, Hawassa University, Hawassa, Ethiopia; 118 Department of Public Health, Ministry of Health (MOH), Hawassa, Ethiopia; 119 Department of Ophthalmology, University of Leipzig Medical Center, Leipzig, Germany; 120 Department of Ophthalmology, Charité Medical University Berlin, Berlin, Germany; 121 College of Nursing, Qatar University, Doha, Qatar; 122 Psychological Sciences Association, Amman, Jordan; 123 Institute of Health Research, University of Health and Allied Sciences, Ho, Ghana; 124 Department of Zoology, Abdul Wali Khan University Mardan, Mardan, Pakistan; 125 Center for Biotechnology and Microbiology, University of SWAT, Swat, Pakistan; 126 Wilmer Eye Institute, Johns Hopkins University School of Medicine, Baltimore, MD USA; 127 Doheny Image Reading and Research Lab (DIRRL), University of California Los Angeles, Los Angeles, CA USA; 128 Department of Pharmacy Practice and Pharmacotherapeutics, University of Sharjah, Sharjah, United Arab Emirates; 129 Department of Clinical Pharmacy, Jordan University of Science and Technology, Irbid, Jordan; 130 Department of Medicine, University of Thessaly, Volos, Greece; 131 Department of Ophthalmology, Inselspital, Bern, Switzerland; 132 Department of Vitreoretinal, Moorfields Eye Hospital, London, UK; 133 Department of Pharmacology, All India Institute of Medical Sciences, Jodhpur, India; 134 All India Institute of Medical Sciences, Bhubaneswar, India; 135 School of Dentistry and Medical Sciences, Charles Sturt University, Orange, NSW Australia; 136 Health Management and Economics Research Center, Iran University of Medical Sciences, Tehran, Iran; 137 Department of Applied Mathematics, University of Washington, Seattle, WA USA; 138 College of Art and Science, Ottawa University, Surprise, AZ USA; 139 School of Life Sciences, Arizona State University, Tempe, AZ USA; 140 Department of Environmental Health, Mekelle University, Mekelle, Ethiopia; 141 Department of Public Health, Jimma University, Jimma, Ethiopia; 142 Department of Public Health, Wachemo University, Hossana, Ethiopia; 143 University Institute of Radiological Sciences and Medical Imaging Technology, The University of Lahore, Lahore, Pakistan; 144 Department of Immunology, Zanjan University of Medical Sciences, Zanjan, Iran; 145 School of Nursing and Midwifery, Debre Berhan University, Debre Berhan, Ethiopia; 146 Faculty of Nursing, Philadelphia University, Amman, Jordan; 147 Department of Forensic Medicine, Lumbini Medical College, Palpa, Nepal; 148 Department of Health Information Management, Iran University of Medical Sciences, Tehran, Iran; 149 Department of Neurovascular Research, Nested Knowledge, Inc., Saint Paul, MN USA; 150 Faculty of Medicine, October 6 University, 6th of October City, Giza Governorate, Egypt; 151 School of Medicine, Isfahan University of Medical Sciences, Isfahan, Iran; 152 School of Public Affairs, Nanjing University of Science and Technology, Nanjing, China; 153 University Institute of Public Health, The University of Lahore, Lahore, Pakistan; 154 Big Data Institute - GRAM Project, University of Oxford, Oxford, UK; 155 Center of Innovation, Technology and Education (CITE), Anhembi Morumbi University, Sao Jose dos Campos, Brazil; 156 Department of Anatomy, Saveh University of Medical Sciences, Saveh, Iran; 157 Department of Medical and Surgical Sciences and Advanced Technologies “GF Ingrassia”, University of Catania, Catania, Italy; 158 Miami Cancer Institute, Baptist Health South Florida, Miami, FL USA; 159 Heidelberg Institute of Global Health (HIGH), Heidelberg University, Heidelberg, Germany; 160 T.H. Chan School of Public Health, Harvard University, Boston, MA USA; 161 Department of Epidemiology, University of Florida, Gainesville, FL USA; 162 Department of Public & Environmental Health, University of The Gambia, Brikama, The Gambia; 163 Department of Ophthalmology, Vita-Salute San Raffaele University, Milan, Italy; 164 Department of Surgery, Jimma University, jimma, Ethiopia; 165 School of Health Science, Bahir Dar University, Bahir Dar, Ethiopia; 166 Department of Pharmacology, Popular Medical College, Dhaka, Bangladesh; 167 Department of Public Health, North Dakota State University, Fargo, ND USA; 168 Department of Community Medicine and Family Medicine, All India Institute of Medical Sciences, Jodhpur, India; 169 School of Public Health, All India Institute of Medical Sciences, Jodhpur, India; 170 Global Health Neurology Lab, NSW Brain Clot Bank, Sydney, NSW Australia; 171 Department of Neurology and Neurophysiology, South West Sydney Local Heath District and Liverpool Hospital, Sydney, NSW Australia; 172 Department of General Medicine, Manipal Academy of Higher Education, Mangalore, India; 173 Medical Lab Technology, Chandigarh University, Mohali, India; 174 Epidemiology Department, Ufa Eye Research Institute, Ufa, Russia; 175 Division of Clinical Epidemiology, McGill University, Montreal, QC Canada; 176 Centre for Outcomes Research and Evaluation, Research Institute of the McGill University Health Centre, Montreal, QC Canada; 177 Department of Biopharmaceutics and Clinical Pharmacy, The University of Jordan, Amman, Jordan; 178 Department of Basic Biomedical Sciences, University of Sharjah, Sharjah, United Arab Emirates; 179 School of Public Health and Health Systems, University of Waterloo, Waterloo, ON Canada; 180 Al Shifa School of Public Health, Al Shifa Trust Eye Hospital, Rawalpindi, Pakistan; 181 Harvard Business School, Harvard University, Boston, MA USA; 182 School of Sciences, University of Minho, Braga, Portugal; 183 Association of Licensed Optometry Professionals, Linda-a-Velha, Portugal; 184 College of Public Health, James Cook University, Townsville, QLD Australia; 185 Department of Public Health, University of Mataram, Mataram, Indonesia; 186 Department of Clinical Pharmacy, University of Gondar, Gondar, Ethiopia; 187 Research School of Population Health, Australian National University, Canberra, ACT Australia; 188 Center for Biomedicine and Community Health, VNU-International School, Hanoi, Vietnam; 189 Therapeutic and Diagnostic Technologies, Cooperativa de Ensino Superior Politécnico e Universitário (Polytechnic and University Higher Education Cooperative), Gandra, Portugal; 190 Institute for Research and Innovation in Health, University of Porto, Porto, Portugal; 191 Department of Addiction Medicine, Haukland University Hospital, Bergen, Norway; 192 Department of Global Public Health and Primary Care, University of Bergen, Bergen, Norway; 193 Public Health Foundation of India, Gurugram, India; 194 Indian Council of Medical Research, New Delhi, India; 195 Ophthalmology Department, Carol Davila University of Medicine and Pharmacy, Bucharest, Romania; 196 Ophthalmology Department, Emergency University Hospital Bucharest, Bucuresti, Romania; 197 dummy2nd University Ophthalmology Department, Aristotle University of Thessaloniki, Thessaloniki, Greece; 198 Ophthalmology Department, University of Thessaly, Larissa, Greece; 199 Department of Medical Biochemistry, University of Gondar, Gondar, Ethiopia; 200 Department of Physiology, Bahir Dar University, Bahir Dar, Ethiopia; 201 Department of Biomedical Sciences, Jimma University, Jimma, Ethiopia; 202 St Paul’s Eye Unit, Royal Liverpool University Hospital, Liverpool, UK; 203 Department of Ophthalmology, Aristotle University of Thessaloniki, Thessaloniki, Greece; 204 Department of Community Medicine, Chettinad Hospital and Research Institute, Chettinad Academy of Research and Education, Chennai, India; 205 Center of Complexity Sciences, National Autonomous University of Mexico, Mexico City, Mexico; 206 Faculty of Veterinary Medicine and Zootechnics, Autonomous University of Sinaloa, Culiacán Rosales, Mexico; 207 Department of Human Physiology, University of Gondar, Gondar, Ethiopia; 208 Department of Medicine, Pham Ngoc Thach University of Medicine, Ho Chi Minh City, Vietnam; 209 Department of Medicine, Can Tho University of Medicine and Pharmacy, Can Tho, Vietnam; 210 Department of Conservative Dentistry with Endodontics, Medical University of Silesia, Katowice, Poland; 211 School of Health Sciences, University of Science Malaysia, Kubang Kerian, Malaysia; 212 Department of Ophthalmology and Visual Sciences, University of Michigan, Ann Arbor, MI USA; 213 Institute for Health Care Policy and Innovation, University of Michigan, Ann Arbor, MI USA; 214 Department of Epidemiology and Medical Statistics, University of Ibadan, Ibadan, Nigeria; 215 Faculty of Public Health, University of Ibadan, Ibadan, Nigeria; 216 Neurophysiology Department, Cairo University, Cairo, Egypt; 217 Faculty of Medicine, University of Tripoli, Tripoli, Libya; 218 Ophthalmic Epidemiology Research Center, Shahroud University of Medical Sciences, Shahroud, Iran; 219 Department of Ophthalmology, University of California Los Angeles, Los Angeles, CA USA; 220 Department of Physical Medicine and Rehabilitation, Johns Hopkins University, Baltimore, MD USA; 221 Research Centre for Healthcare and Community, Coventry University, Coventry, UK; 222 School of Medicine, Tehran University of Medical Sciences, Tehran, Iran; 223 Endocrinology and Metabolism Research Institute, Non-Communicable Diseases Research Center, Tehran, Iran; 224 Department of Environmental Health Engineering, Isfahan University of Medical Sciences, Isfahan, Iran; 225 Department of Social Medicine and Epidemiology, Guilan University of Medical Sciences, Rasht, Iran; 226 University Eye Clinic, University of Genoa, Genoa, Italy; 227 Department of Nursing, Wollega University, Nekemte, Ethiopia; 228 Institute of Public Health, Charité Medical University Berlin, Berlin, Germany; 229 Department of Ophthalmology, Isfahan University of Medical Sciences, Isfahan, Iran; 230 Emergency Department, Isfahan University of Medical Sciences, Isfahan, Iran; 231 Division of Ophthalmology, University of São Paulo, Ribeirão Preto, Brazil; 232 Community Medicine Department, Bayero University, Kano, Kano, Nigeria; 233 Department of Community Medicine, Aminu Kano Teaching Hospital, Kano, Nigeria; 234 Department of Community Medicine, Datta Meghe Institute of Medical Sciences, Wardha, India; 235 Department of Community Medicine, ESIC Medical College & Hospital, Hyderabad, India; 236 Health Sciences Department of Oncology Nursing, Haramaya University, Harar, Ethiopia; 237 Department of Environmental Health, Wollo University, Dessie, Ethiopia; 238 Department of Nursing, Aksum University, Aksum, Ethiopia; 239 Department of Nursing, Mekelle University, Mekelle, Ethiopia; 240 Department of Neurology, Tehran University of Medical Sciences, Tehran, Iran; 241 Eye Research Center, Iran University of Medical Sciences, Tehran, Iran; 242 Ophthalmology Department, Tehran University of Medical Sciences, Tehran, Iran; 243 Department of Radiology, Mayo Clinic, Rochester, MN USA; 244 Department of Applied Cell Sciences, Urmia University of Medical Sciences, Urmia, Iran; 245 Cellular and Molecular Medicine Institute, Urmia University of Medical Sciences, Urmia, Iran; 246 Health Systems and Policy Research Department, Indian Institute of Public Health, Gandhinagar, India; 247 Department of Genetics, Sana Institute of Higher Education, Sari, Iran; 248 Universal Scientific Education and Research Network (USERN), Kermanshah University of Medical Sciences, Kermanshah, Iran; 249 Department of Epidemiology and Biostatistics, Anhui Medicla University, Hefei, China; 250 Toxicology Department, Shriram Institute for Industrial Research, Delhi, India; 251 Faculty of Medicine Health and Human Sciences, Macquarie University, Sydney, NSW Australia; 252 Department of Pharmacology and Toxicology, Hamadan University of Medical Sciences, Hamadan, Iran; 253 Brain and Behavioral Sciences Program, University of Georgia, Athens, GA USA; 254 Department of Nursing, Arak University of Medical Sciences, Arak, Iran; 255 Department of Zoology and Entomology, Al Azhar University, Cairo, Egypt; 256 Department of Pharmaceutical Technology, University of Dhaka, Dhaka, Bangladesh; 257 Department of Ophthalmology, Iran University of Medical Sciences, Karaj, Iran; 258 Social Determinants of Health Research Center, Shahid Beheshti University of Medical Sciences, Tehran, Iran; 259 Chapter of Addiction Medicine, University of Sydney, Sydney, NSW Australia; 260 Independent Consultant, Santa Clara, CA USA; 261 Department of Public Health, Madda Walabu University, Robe, Ethiopia; 262 Kasturba Medical College, Mangalore, Manipal Academy of Higher Education, Manipal, India; 263 Institute of Research and Development, Duy Tan University, Da Nang, Vietnam; 264 Department of Computer Science, University of Human Development, Sulaymaniyah, Iraq; 265 Department of Psychology, Tsinghua University, Beijing, China; 266 School of Biotechnology, Tan Tao University, Long An, Vietnam; 267 Department of Occupational Safety and Health, China Medical University, Taichung, Taiwan; 268 Department of Occupational Therapy, Asia University, Taiwan, Taichung, Taiwan; 269 Department of Public Health, University of Naples Federico II, Naples, Italy; 270 Faculty of Medicine, University of Belgrade, Belgrade, Serbia; 271 Institute for Physical Activity and Nutrition, Deakin University, Burwood, VIC Australia; 272 Sydney Medical School, University of Sydney, Sydney, NSW Australia; 273 Research and Development Unit, Biomedical Research Networking Center for Mental Health Network (CiberSAM), Sant Boi de Llobregat, Spain; 274 Faculty of Medicine, University of Versailles Saint-Quentin-en-Yvelines, Montigny-le-Bretonneux, France; 275 Department of Immunology, Kerman University of Medical Sciences, Kerman, Iran; 276 Department of Immunology, Rafsanjan University of Medical Sciences, Rafsanjan, Iran; 277 Institute of Advanced Manufacturing Technologies, Peter the Great St. Petersburg Polytechnic University, St, Petersburg, Russia; 278 Institute of Comparative Economic Studies, Hosei University, Tokyo, Japan; 279 Manipal College of Pharmaceutical Sciences, Manipal Academy of Higher Education, Manipal, India; 280 Centre of Studies and Research, Ministry of Health, Muscat, Oman; 281 Department of Biochemistry, Government Medical College, Mysuru, India; 282 Institute of Molecular and Clinical Ophthalmology Basel, Basel, Switzerland; 283 Department of Ophthalmology, Heidelberg University, Mannheim, Germany; 284 Department of Community Medicine, Manipal Academy of Higher Education, Mangalore, India; 285 Department of Economics, National Open University, Benin City, Nigeria; 286 Manipal Institute of Management, Manipal Academy of Higher Education, Manipal, India; 287 Save Sight Institute, University of Sydney, Sydney, NSW Australia; 288 Sydney Eye Hospital, South Eastern Sydney Local Health District, Sydney, NSW Australia; 289 School of Health Professions and Human Services, Hofstra University, Hempstead, NY USA; 290 Department of Anesthesiology, Montefiore Medical Center, Bronx, NY USA; 291 Health Policy Research Center, Shiraz University of Medical Sciences, Shiraz, Iran; 292 Department of Ophthalmology, Yenepoya Medical College, Mangalore, India; 293 Public Health Foundation of India, New Delhi, India; 294 Department of ENT, Dr. B. R. Ambedkar State Institute of Medical Sciences (AIMS), Mohali, India; 295 International Research Center of Excellence, Institute of Human Virology Nigeria, Abuja, Nigeria; 296 Julius Centre for Health Sciences and Primary Care, Utrecht University, Utrecht, Netherlands; 297 Department of Public Health, Jordan University of Science and Technology, Irbid, Jordan; 298 Amity Institute of Forensic Sciences, Amity University, Noida, India; 299 Department of Biophysics and Biochemistry, Baku State University, Baku, Azerbaijan; 300 Azerbaijan State University of Economics (UNEC), Baku, Azerbaijan; 301 Natural and Medical Sciences Research Center, University of Nizwa, Oman, Nizwa, Oman; 302 Department of Basic Medical Sciences, Yarmouk University, Irbid, Jordan; 303 Global Consortium for Public Health Research, Datta Meghe Institute of Higher Education and Research, Wardha, India; 304 Department of Medical Physiology, Bahir Dar University, Bahir Dar, Ethiopia; 305 School of Traditional Chinese Medicine, Xiamen University Malaysia, Sepang, Malaysia; 306 School of Health Sciences, Kristiania University College, Oslo, Norway; 307 Department of International Health and Sustainable Development, Tulane University, New Orleans, LA USA; 308 Department of Nursing and Health Promotion, Oslo Metropolitan University, Oslo, Norway; 309 Independent Consultant, Jakarta, Indonesia; 310 San Juan de Dios Sanitary Park, Barcelona, Spain; 311 Department of Anthropology, Panjab University, Chandigarh, India; 312 Faculty of Medicine, Gazi University, Ankara, Türkiye; 313 Department of General Surgery, Dr NTR University of Health Sciences, Vijayawada, India; 314 Department of Health Policy and Strategy, Foundation for People-centric Health Systems, New Delhi, India; 315 SD Gupta School of Public Health, Indian Institute of Health Management Research University, Jaipur, India; 316 Department of Physiotherapy, Universitas Aisyiyah Yogyakarta, Yogyakarta, Indonesia; 317 Institute of Allied Health Sciences, National Cheng Kung University, Tainan, Taiwan; 318 Chief Medical Office, HelpMeSee, New York, NY USA; 319 Mexican Institute of Ophthalmology, Queretaro, Mexico; 320 Department of Medical Science, Ajou University School of Medicine, Suwon, South Korea; 321 Department of Precision Medicine, Sungkyunkwan University, Suwon, South Korea; 322 Department of Internal Medicine, University of Texas, Galveston, TX USA; 323 Lerner Research Institute, Cleveland Clinic, Cleveland, OH USA; 324 Department of Quantitative Health Science, Case Western Reserve University, Cleveland, OH USA; 325 School of Pharmacy, University of the West Indies, St. Augustine, Trinidad and Tobago; 326 Fellow, Planetary Health Alliance, Boston, MA USA; 327 Department of Ophthalmology, Tehran University of Medical Sciences, Tehran, Iran; 328 Department of Internal Medicine, Dayanand Medical College and Hospital, Ludhiana, India; 329 Rabigh Faculty of Medicine, King Abdulaziz University, Jeddah, Saudi Arabia; 330 Electrical Engineering Department, Prince Sattam bin Abdulaziz University, Al Kharj, Saudi Arabia; 331 Department of Clinical Pharmacy, Jouf University, Sakaka, Saudi Arabia; 332 Digestive Diseases Research Institute, Tehran University of Medical Sciences, Tehran, Iran; 333 Department of Public Health, Management and Science University, Shah Alam, Malaysia; 334 Jeffrey Cheah School of Medicine and Health Sciences, Monash University, Subang Jaya, Malaysia; 335 School of Public Health, Mekelle University, Mekelle, Ethiopia; 336 Department of Nursing, Arba Minch University, Arba Minch, Ethiopia; 337 University Centre Varazdin, University North, Varazdin, Croatia; 338 Pacific Institute for Research & Evaluation, Calverton, MD USA; 339 School of Public Health, Curtin University, Perth, WA Australia; 340 Macquarie Medical School, Macquarie University, Sydney, NSW Australia; 341 National Data Management Center for Health, Ethiopian Public Health Institute, Addis Ababa, Ethiopia; 342 Department of Surgical Oncology, All India Institute of Medical Sciences, Jodhpur, India; 343 Department of Epidemiology and Biostatistics, Shahrekord University of Medical Sciences, Shahrekord, Iran; 344 Translational Ophthalmology Research Center, Tehran University of Medical Sciences, Tehran, Iran; 345 Department of Pharmacology, Abadan School of Medical Sciences, Abadan, Iran; 346 Department of Optometry and Vision Sciences, Zahedan University of Medical Sciences, Zahedan, Iran; 347 Eye Research Center, Mashhad University of Medical Sciences, Mashhad, Iran; 348 Non-Communicable Diseases Research Center, Tehran University of Medical Sciences, Tehran, Iran; 349 School of Medicine, Shahid Beheshti University of Medical Sciences, Tehran, Iran; 350 Iran University of Medical Sciences, Tehran, Iran; 351 College of Medicine and Public Health, Flinders University, Adelaide, Australia; 352 Department of Engineering, Western Sydney University, Sydney, NSW Australia; 353 Comprehensive Cancer Center, University of Alabama at Birmingham, Birmingham, AL USA; 354 Department of Dental Public Health, King Abdulaziz University, Jeddah, Saudi Arabia; 355 Department of Health Policy and Oral Epidemiology, Harvard University, Boston, MA USA; 356 Department of Biotechnology, University of Central Punjab, Lahore, Pakistan; 357 Department of Medical Laboratory Sciences, Adigrat University, Adigrat, Ethiopia; 358 Department of Epidemiology, Non-Communicable Diseases Research Center, Tehran, Iran; 359 Division of Cardiology, Massachusetts General Hospital, Boston, MA USA; 360 Department of Medical Engineering, University of South Florida, Tampa, FL USA; 361 Cardiovascular Laboratory, Methodist Hospital, Merrillville, Merrillville, IN USA; 362 Department of Allergy, Immunology and Dermatology, Hanoi Medical University, Hanoi, Vietnam; 363 Cardiovascular Research Department, Methodist Hospital, Merrillville, IL USA; 364 Department of Surgery, Danang Family Hospital, Danang, Vietnam; 365 Department of General Medicine, University of Medicine and Pharmacy at Ho Chi Minh City, Ho Chi Minh City, Vietnam; 366 International Islamic University Islamabad, Islamabad, Pakistan; 367 Department of Applied Microbiology, Oslo University Hospital, Taiz, Yemen; 368 Faculty of Applied Sciences and Technology, Universiti Tun Hussein Onn Malaysia, Johor, Malaysia; 369 Department of Applied Economics and Quantitative Analysis, University of Bucharest, Bucharest, Romania; 370 School of Pharmacy, University of the Western Cape, Cape Town, South Africa; 371 Department of Psychiatry and Behavioural Neurosciences, McMaster University, Hamilton, ON Canada; 372 Department of Psychiatry, University of Lagos, Lagos, Nigeria; 373 Slum and Rural Health Initiative Research Academy, Slum and Rural Health Initiative, Ibadan, Nigeria; 374 Department of Pharmacology and Therapeutics, University of Nigeria Nsukka, Enugu, Nigeria; 375 Geography Department, East Carolina University, Greenville, NC USA; 376 Department of Pharmacotherapy and Pharmaceutical Care, Medical University of Warsaw, Warsaw, Poland; 377 School of Medicine, Western Sydney University, Campbelltown, NSW Australia; 378 Department of Optometry and Vision Science, University of KwaZulu-Natal, KwaZulu-Natal, South Africa; 379 Laboratory of Public Health Indicators Analysis and Health Digitalization, Moscow Institute of Physics and Technology, Dolgoprudny, Russia; 380 Department of Medicine, University of Ibadan, Ibadan, Nigeria; 381 Department of Medicine, University College Hospital, Ibadan, Nigeria; 382 Department of Forensic Medicine and Toxicology, Kasturba Medical College, Mangalore, Mangalore, India; 383 Privatpraxis, Heidelberg, Germany; 384 Global Health Governance Programme, University of Edinburgh, Edinburgh, UK; 385 School of Dentistry, University of Leeds, Leeds, UK; 386 Department of Genetics, Yale University, New Haven, CT USA; 387 Department of Development Studies, International Institute for Population Sciences, Mumbai, India; 388 Department of Zoology, Yadava College, Madurai, India; 389 Department of Zoology, Annai Fathima College, Madurai, India; 390 Department of Statistics and Econometrics, Bucharest University of Economic Studies, Bucharest, Romania; 391 Medical School, Pham Ngoc Thach University of Medicine, Ho Chi Minh City, Vietnam; 392 Department of Maternal and Child Nursing and Public Health, Federal University of Minas Gerais, Belo Horizonte, Brazil; 393 Department of Neonatology, Case Western Reserve University, Cleveland, OH USA; 394 Manipal TATA Medical College, Manipal Academy of Higher Education, Manipal, India; 395 Department of Population Science and Human Resource Development, University of Rajshahi, Rajshahi, Bangladesh; 396 Department of Radiology, Stanford University School of Medicine, Stanford, CA USA; 397 Department of Community Medicine, Mahatma Gandhi Medical College and Research Institute, Puducherry, India; 398 Department Biological Sciences, King Abdulaziz University, Jeddah, Egypt; 399 Department of Protein Research, Research and Academic Institution, Alexandria, Egypt; 400 Health Economics Research Centre, University of Oxford, Oxford, UK; 401 Department of Pharmacology and Toxicology, University of Antioquia, Medellin, Colombia; 402 Faculty of Medicine, Gonabad University of Medical Sciences, Gonabad, Iran; 403 Infectious Diseases Research Center, Gonabad University of Medical Sciences, Gonabad, Iran; 404 Department of Epidemiology, Shahid Beheshti University of Medical Sciences, Tehran, Iran; 405 Sharjah Institute for Medical Research, University of Sharjah, Sharjah, United Arab Emirates; 406 Multidisciplinary Laboratory Foundation University School of Health Sciences (FUSH), Foundation University, Islamabad, Pakistan; 407 International Center of Medical Sciences Research (ICMSR), Islamabad, Pakistan; 408 Ophthalmic Epidemiology Research Center, Shahid Beheshti University of Medical Sciences, Tehran, Iran; 409 Ophthalmic Research Center, Shahid Beheshti University of Medical Sciences, Tehran, Iran; 410 Research Center for Immunodeficiencies, Tehran University of Medical Sciences, Tehran, Iran; 411 Sharjah Institute of Medical Sciences, University of Sharjah, Sharjah, United Arab Emirates; 412 Clinical Sciences Department, Sharjah, United Arab Emirates; 413 Applied Biomedical Research Center, Mashhad University of Medical Sciences, Mashhad, Iran; 414 Biotechnology Research Center, Mashhad University of Medical Sciences, Mashhad, Iran; 415 Multiple Sclerosis Research Center, Tehran University of Medical Sciences, Tehran, Iran; 416 Ludwig Maximilian University of Munich, Munich, Germany; 417 Institute for Employment Research, Nuremberg, Germany; 418 College of Medicine, University of Sharjah, Sharjah, United Arab Emirates; 419 Faculty of Pharmacy, Mansoura University, Mansoura, Egypt; 420 Department of Neurology, Charité University Medical Center Berlin, Berlin, Germany; 421 Department of Neurology, University of Southern Denmark, Odense, Denmark; 422 School of Public Health, Taipei Medical University, Taipei, Taiwan; 423 Department of Entomology, Ain Shams University, Cairo, Egypt; 424 Medical Ain Shams Research Institute (MASRI), Ain Shams University, Cairo, Egypt; 425 Market Access, Bayer, Istanbul, Türkiye; 426 Faculty of Dentistry, AIMST University, Bedong, Malaysia; 427 Department of Medicine and Surgery, Government Doon Medical College, Dehradun, India; 428 National Heart, Lung, and Blood Institute, National Institute of Health, Rockville, MD USA; 429 Department of Clinical Sciences, Al-Quds University, Ajman, United Arab Emirates; 430 Independent Consultant, Karachi, Pakistan; 431 Department of Nursing, Debre Berhan University, Debre Berhan, Ethiopia; 432 National Institute of Infectious Diseases, Tokyo, Japan; 433 Department of Veterinary Public Health and Preventive Medicine, Usmanu Danfodiyo University, Sokoto, Sokoto, Nigeria; 434 Department of International Studies, Non-Communicable Diseases Research Center, Tehran, Iran; 435 Faculty of Medicine, Tehran University of Medical Sciences, Tehran, Iran; 436 Department of Medical-Surgical Nursing, Mazandaran University of Medical Sciences, Sari, Iran; 437 Department of Nursing and Health Sciences, Flinders University, Adelaide, Australia; 438 Department of Pediatrics and Child Health Nursing, Dilla University, Dilla, Ethiopia; 439 Unit of Basic Medical Sciences, University of Khartoum, Khartoum, Sudan; 440 Department of Medical Microbiology and Infectious Diseases, Erasmus University, Rotterdam, Netherlands; 441 Family, Health Promotion, and Life Course Department, Pan American Health Organization, Bogota, Colombia; 442 School of Medicine, University of Alabama at Birmingham, Birmingham, AL USA; 443 Department of Medicine Service, US Department of Veterans Affairs (VA), Birmingham, AL USA; 444 Department of Radiodiagnosis, All India Institute of Medical Sciences, Bathinda, India; 445 Department of Radiology, University of Alabama at Birmingham, Birmingham, AL USA; 446 Directive Board, Association of Licensed Optometry Professionals, Linda-a-Velha, Portugal; 447 Division of Community Medicine, International Medical University, Kuala Lumpur, Malaysia; 448 Department of Pharmacology, Shaqra University, Shaqra, Saudi Arabia; 449 Trauma and Injury Research Center, Iran University of Medical Sciences, Tehran, Iran; 450 Medical Ethics and Law Research Center, Shahid Beheshti University of Medical Sciences, Tehran, Iran; 451 Aier Eye Hospital, Jinan University, Guangzhou, China; 452 Pediatric Intensive Care Unit, King Saud University, Riyadh, Saudi Arabia; 453 Faculty of Public Health, Universitas Sam Ratulangi, Manado, Indonesia; 454 Nuffield Department of Primary Care Health Sciences, University of Oxford, Oxford, UK; 455 Department of Public Health, Woldia University, Woldia, Ethiopia; 456 Department of Medicine, University of Crete, Heraklion, Greece; 457 Medical School, University of Crete, Heraklion, Greece; 458 Department of Pharmacology, All India Institute of Medical Sciences, Deoghar, India; 459 Department of Public Health, East Carolina University, Greenville, NC USA; 460 College of Public Health, Temple University, Philadelphia, PA USA; 461 Medical Genomics Research Department, King Abdullah International Medical Research Center, Riyadh, Saudi Arabia; 462 Department of Life Sciences, University of Management and Technology, Lahore, Pakistan; 463 Clinical Cancer Research Center, Milad General Hospital, Tehran, Iran; 464 Department of Microbiology, Islamic Azad University, Tehran, Iran; 465 Urmia University of Medical Sciences, Urmia, Iran; 466 Division of Cardiology, Johns Hopkins University, Baltimore, MD USA; 467 Department of Epidemiology and Biostatistics, Bahir Dar University, Bahir Dar, Ethiopia; 468 Department of Community Medicine, Rajarata University of Sri Lanka, Anuradhapura, Sri Lanka; 469 Department of Ophthalmology Research, Queen Mamohato Memorial Hospital, Maseru, Lesotho; 470 Ophthalmology Unit, Bahir Dar University, Bahir Dar, Ethiopia; 471 Department of Microbiology and Immunology, Zagazig University, Zagazig, Egypt; 472 Department of Cells and Tissues, Molecular Biology Institute of Barcelona, Barcelona, Spain; 473 Department of Cancer Epidemiology and Prevention Research, Alberta Health Services, Calgary, AB Canada; 474 Department of Oncology, University of Calgary, Calgary, AB Canada; 475 China Center for Health Development Studies, Peking University, Beijing, China; 476 Center for the Study of Aging and Human Development, Duke University, Durham, NC USA; 477 Department of Health Management, Süleyman Demirel Üniversitesi (Süleyman Demirel University), Isparta, Türkiye; 478 Department of Pharmacology, Bahir Dar University, Bahir Dar, Ethiopia; 479 Pharmacy Department, Alkan Health Science Business and Technology College, Bahir Dar, Ethiopia; 480 Department of Neuropsychopharmacology, National Center of Neurology and Psychiatry, Kodaira, Japan; 481 Department of Public Health, Juntendo University, Tokyo, Japan; 482 Department of Bioengineering and Therapeutic Sciences, University of California San Francisco, San Francisco, CA USA; 483 Addictology Department, Russian Medical Academy of Continuous Professional Education, Moscow, Russia; 484 Department of Public Health, Dilla University, Dilla, Ethiopia; 485 School of Medicine, Wuhan University, Wuhan, China; 486 College of Traditional Chinese Medicine, Hebei University, Baoding, China; 487 Department of Biochemistry and Pharmacogenomics, Medical University of Warsaw, Warsaw, Poland; 488 Department of Nursing, Yasuj University of Medical Sciences, Yasuj, Iran

**Keywords:** Epidemiology, Public health

## Abstract

**Background:**

Uncorrected refractive error (URE) is a readily treatable cause of visual impairment (VI). This study provides updated estimates of global and regional vision loss due to URE, presenting temporal change for VISION 2020

**Methods:**

Data from population-based eye disease surveys from 1980–2018 were collected. Hierarchical models estimated prevalence (95% uncertainty intervals [UI]) of blindness (presenting visual acuity (VA) < 3/60) and moderate-to-severe vision impairment (MSVI; 3/60 ≤ presenting VA < 6/18) caused by URE, stratified by age, sex, region, and year. Near VI prevalence from uncorrected presbyopia was defined as presenting near VA < N6/N8 at 40 cm when best-corrected distance (VA ≥ 6/12).

**Results:**

In 2020, 3.7 million people (95%UI 3.10–4.29) were blind and 157 million (140–176) had MSVI due to URE, a 21.8% increase in blindness and 72.0% increase in MSVI since 2000. Age-standardised prevalence of URE blindness and MSVI decreased by 30.5% (30.7–30.3) and 2.4% (2.6–2.2) respectively during this time. In 2020, South Asia GBD super-region had the highest 50+ years age-standardised URE blindness (0.33% (0.26–0.40%)) and MSVI (10.3% (8.82–12.10%)) rates. The age-standardized ratio of women to men for URE blindness was 1.05:1.00 in 2020 and 1.03:1.00 in 2000. An estimated 419 million (295–562) people 50+ had near VI from uncorrected presbyopia, a +75.3% (74.6–76.0) increase from 2000

**Conclusions:**

The number of cases of VI from URE substantively grew, even as age-standardised prevalence fell, since 2000, with a continued disproportionate burden by region and sex. Global population ageing will increase this burden, highlighting urgent need for novel approaches to refractive service delivery.

## Introduction

Uncorrected refractive error (URE) is the leading cause of vision impairment globally among both adults and children, and contributes to reduced educational and economic opportunities [1–6], decreased quality of life [7] and an increased burden of mortality [8–10]. Visual impairment is a significant global health concern and the financial burden with global productivity losses is estimated to be 411 billion US dollars annually [11]. URE is readily treated with spectacles, making it one of the most cost-effective healthcare interventions, alongside cataract surgery [12–15]. Thus, it is a global priority to improve access to refraction services [11], as set out in ‘Towards universal eye health: Global Action Plan 2014–2019 of the World Health Assembly (WHA) in 2013 [16]. and more recently in the ‘World Report on Vision’ by the World Health Organisation (WHO) in 2019 [17], which called for the routine measurement of refractive error services coverage as a means to address the United Nations (UN) Sustainable Development Goals [18] target 3.8 to “achieve universal health coverage, including financial risk protection, access to quality essential healthcare services and access to safe, effective, quality and affordable essential medicines and vaccines for all”. Furthermore, these recommendations have been adopted in a resolution by the 73rd WHA member states in 2021, which set global targets for a 40% increase in effective refractive error coverage (eREC) by 2030. As we transition from the efforts of VISION 2020: the Right to Sight initiative to tackle avoidable blindness, these focused targets are fundamental to eliminate avoidable vision loss in future.

Refractive error is a common ocular condition which occurs throughout the lifespan [19], and chiefly falls into the following categories: myopia (affecting mostly distance vision), hyperopia (potentially causing impaired vision at distance and near), which may both be accompanied by an astigmatic component, and presbyopia (characterised by poor near vision). The last 20 years have seen rapid increases in the prevalence of myopia across the world, particularly in East Asia [20–23].

Nearly all individuals, even those without significant refractive error in childhood and earlier adult years will acquire presbyopia by the 5th decade of life, necessitating refractive correction for near work. Presbyopia occurs due to reduced flexibility of the human crystalline lens to accommodate (focus) on near targets, resulting in blurred near vision. It is an essentially universal phenomenon, with age of onset determined by factors such as the presence of latent hyperopia. Without refractive correction, vision deteriorates for near activities, resulting in near visual impairment. Where uncorrected hyperopia is common, due to lack of access to both education (which induces myopia [24]) and refractive services, the onset of presbyopia may even occur in the 30 s, which is a critical working age for those in industries such as garments and textiles [25, 26]. With an ageing global population, the burden of presbyopia and near visual impairment will increase. Coupled with the impact of the increased prevalence of myopia, the burden of URE is likely to grow in the future.

The Vision Loss Expert Group (VLEG) curate a comprehensive, continuously updated, online database of ophthalmic epidemiological data and have made important contributions to knowledge about the burden and causes of vision impairment and blindness globally [27–29]. These estimates have been used in the WHO Report on Vision in 2019 [17] and the recent Lancet Global Health commission on Global Eye Health Report [15]. Updated analyses are required to reflect rapidly increasing sources of new population data, and to monitor progress in reduction of avoidable sight loss. The need for new population data on vision impairment has been emphasised in a recent paper highlighting the grand challenge priorities for global eye health [30], and will be vital to monitor and measure success against the WHA global target of a 40% increase in eREC.

Thus, the aim of the current study is to provide updated estimates of the global burden of vision loss due to URE, disaggregated by sex, age, year and region, for the period from 2000 to 2020 covered by VISION 2020: The Right to Sight initiative. For the first time, temporal trends will be calculated to present the burden of visual impairment resulting from uncorrected presbyopia in those 50+ years.

## Methods

A systematic review of population-based studies of vision impairment and blindness published between Jan 1, 1980, and Oct 1, 2018, was carried out, which included grey literature sources. Eligible studies from this review were then combined with data from Rapid Assessment of Avoidable Blindness (RAAB) studies and finally, data from the US National Health and Nutrition Examination Survey and the WHO Study on Global Ageing and Adult Health were added. More detailed methods are published elsewhere [30, 31], and outlined below.

In total, the VLEG review identified 243 studies (73% were rapid studies) across 73 countries from which data relating to the contribution of URE to vision loss could be extracted: with 70 studies from the 2010 review [28], and a further 173 studies in an extension of the literature review to 2018 [29]. Studies were primarily national and subnational cross-sectional surveys. By the seven World Global Burden of Disease (GBD) super regions, 43 studies were from Sub-Saharan Africa, 100 from Southeast Asia, East Asia, and Oceania, 44 from South Asia, 16 from North Africa and the Middle East, 25 from Latin America and the Caribbean, 9 from High income, and 6 from Central Europe, Eastern Europe, and Central Asia. Additionally, the VLEG commissioned the preparation of 5-year age-disaggregated RAAB data from the RAAB repository. Studies were included if they met the following criteria: visual acuity data had to be measured using a test chart that could be mapped to the Snellen scale, and the sample had to be representative of the population. Studies based on self-report of vision loss were excluded. The International Classification of Diseases 11th edition [32] criteria for vision loss, as suggested by WHO, was employed, categorizing individuals according to vision in their better eye on presentation. This classification defines moderate vision loss as visual acuity of 6/60 or better but less than 6/18, severe vision loss as a visual acuity of 3/60 or better but less than 6/60, and blindness as visual acuity of less than 3/60 or less than 10° visual field around central fixation (although the visual field definition is rarely used in population-based eye surveys). Moderate and severe visual impairment (MSVI) was combined to present prevalence data. Vision impairment from uncorrected presbyopia was defined as presenting near vision of worse than <N6 or <N8 at 40 cm where best-corrected distance visual acuity was 6/12 or better. Prevalence of near VI from uncorrected presbyopia was based on 25 studies.

The global and regional prevalence and burden of blindness and MSVI due to URE were gathered from the all-cause meta-analysis and modelling. First, we separated raw data into datasets including so-called vision loss envelopes (see Flaxman et al. [28]. for explanation) for all-cause mild, moderate, and severe vision loss, and blindness. Data were input into a mixed-effects meta-regression tool developed by the Institute for Health Metrics and Evaluation and called MR-BRT (meta-regression; Bayesian; regularised; trimmed) [33]. Presenting vision impairment was the reference definition for each level of severity. Prevalence data for URE were extracted directly where available and otherwise calculated by subtracting best-corrected vision impairment from presenting vision impairment prevalence for each level of severity in studies that reported both measures for a given location, sex, age group, and year. All other causes were quantified as part of the best-corrected estimates of vision impairment at each level of severity.

We modelled distance vision impairment and blindness attributable to the following causes: cataract, URE, age-related macular degeneration, myopic macular degeneration, glaucoma, diabetic retinopathy, and other causes of vision impairment (in aggregate).

We produced location-, year-, age-, and sex-specific estimates of MSVI and blindness using Disease Modelling Meta-Regression (Dismod-MR) 2.1 [34]. The details of the data processing steps are described elsewhere [29]. Briefly, Dismod-MR 2.1 models were run for all vision impairment stratified by severity (moderate, severe, blindness) regardless of cause and, separately, for MSVI and blindness due to each modelled cause of vision impairment. Then, models of MSVI due to specific causes were split into moderate and severe vision loss estimates using the ratio of overall prevalence in the all-cause moderate presenting vision impairment and severe presenting vision impairment models. Next, prevalence estimates for all causes stratified by severity were scaled to the models of all-cause prevalence by severity. This produced final estimates by age, sex, year, and location for each individual cause of vision impairment stratified by severity, including refractive error. Model projection was to the year 2020, coincident with the end of VISION 2020: the Right to Sight initiative, and estimates were age-standardised using the GBD standard population [35]. All generated estimates for visual impairment due to URE are accompanied by 95% uncertainty intervals (UI), which represent the 25th and 975th ordered estimates of 1000 draw estimates of the posterior distribution. We considered estimates to be significantly different if the 95% UIs did not overlap. Data are presented for the total population and also for individuals aged 50+ years, as data sources such as RAAB surveys are major sources of data for low-income and low- or middle-income countries (LICs and LMICs) and these surveys are conducted on individuals aged 50 years and older. The data estimates reported in this study were produced in compliance with the Guidelines for Accurate and Transparent Health Estimates Reporting [36].

Data are presented for the seven World super-regions based on the GBD regional classification system [37], and sub-divided into the 21 GBD world regions. These seven super regions are drawn together based on two criteria: epidemiological similarity and geographic proximity.

## Results

We used 243 data sources from 73 countries to calculate the global and regional prevalence and burden of blindness and MSVI due to URE. Table 1 presents the number of people, men and women, with blindness (<3/60) or MSVI (<6/18 to >/=3/60) due to URE in 2020 in the seven super-regions based on the GBD classification system. Appendix 1 contains supplementary tables for all 21 GBD world regions in 2020. These estimates reveal that in 2020, 3.70 million people (95% UI 3.10–4.29 million) in the world were blind and 157 million (95% UI 140–175 million) had MSVI due to URE. Focusing on those 50+ years of age, 2.29 million people (95% UI 1.79–2.80 million) were blind due to URE globally and 86.1 million (95% UI 74.2–101 million) had MSVI.

**Table 1:** Number and age-standardised prevalence of people with blindness (<3/60) or MSVI (<6/18 to >/=3/60) due to URE in 7 Super Regions in 2020.

	GLOBAL	Central Europe, Eastern Europe, and Central Asia	High income	Latin America and Caribbean	North Africa and Middle East	South Asia	Southeast Asia, East Asia, and Oceania	Sub-Saharan Africa
Total population 2020 (thousands)	7,890,000	417,000	1,090,000	602,000	632,000	1,840,000	2,190,000	1,110,000
**Number of people with blindness (thousands)**	**3700 (3100**–**4290)**	**29.4 (23.3**–**36.0)**	**80.0 (63.6**–**97.6)**	**218 (180**–**258)**	**190 (156**–**229)**	**1520 (1260**–**1770)**	**1410 (1150**–**1650)**	**254 (212**–**303)**
Number of Men with blindness (thousands)	1750 (1480–2020)	13.7 (10.8–17.1)	36.9 (29.1–44.8)	96.1 (78.6–114)	90.4 (73.6–109)	728 (606 –847)	657 (539–769)	123 (102–146)
Number of Women with blindness (thousands)	1950 (1620–2280)	15.7 (12.4–19.2)	43.1 (34.6–52.3)	122 (101–144)	99.8 (81.8–120)	789 (658–925)	750 (616–879)	132 (110–157)
**Number of people with blindness aged 50+ years (thousands)**	**2290 (1790**–**2800)**	**16.8 (12.8**–**21.1)**	**46.1 (35.7**–**56.9)**	**126 (97.5**–**153)**	**84.2 (63.8**–**103)**	**976 (762**–**1190)**	**933 (728**–**1140)**	**111 (84.5**–**136)**
Number of Men with blindness aged 50+ years (thousands)	1050 (816–1270)	7.05 (5.27–8.88)	21.1 (16.2–26.1)	53.8 (41.3–65.7)	39.3 (29.6–48.3)	459 (358–558)	415 (325–509)	52.5 (40.2–65.0)
Number of Women with blindness aged 50+ years (thousands)	1250 (975 –1520)	9.76 (7.35–12.4)	25.0 (19.3–30.9)	72.5 (56.4–87.6)	44.9 (34.1–55.3)	517 (402–633)	518 (406–632)	58.2 (44.3–71.9)
**Age-standardised prevalence of blindness**	**0.04% (0.04**–**0.05)**	**0.01% (0.00**–**0.01)**	**0.01% (0.00**–**0.01)**	**0.04% (0.03**–**0.04)**	**0.03% (0.03**–**0.04)**	**0.10% (0.08**–**0.12)**	**0.05% (0.04**–**0.06)**	**0.04% (0.03**–**0.04)**
Age-standardised prevalence of blindness: Men	0.04% (0.04–0.05)	0.01% (0.00–0.01)	0.01% (0.00–0.01)	0.03% (0.03–0.04)	0.03% (0.03–0.04)	0.10% (0.08–0.11)	0.05% (0.04–0.06)	0.04% (0.03–0.05)
Age-standardised prevalence of blindness: Women	0.05% (0.04–0.05)	0.01% (0.00–0.01)	0.01% (0.00–0.01)	0.04% (0.03–0.04)	0.04% (0.03–0.04)	0.10% (0.09–0.12)	0.06% (0.05–0.06)	0.04% (0.03–0.04)
**Age-standardised prevalence of blindness 50+ years**	**0.12% (0.10**–**0.15)**	**0.01% (0.01**–**0.02)**	**0.01% (0.01**–**0.01)**	**0.09% (0.07**–**0.11)**	**0.09% (0.07**–**0.11)**	**0.33% (0.26**–**0.40)**	**0.15% (0.12**–**0.18)**	**0.11% (0.09**–**0.14)**
Age-standardised prevalence of blindness in Men 50+ years	0.12% (0.09–0.14)	0.01% (0.01–0.02)	0.01% (0.01–0.01)	0.09% (0.07–0.11)	0.08% (0.06–0.10)	0.32% (0.25–0.38)	0.14% (0.11–0.17)	0.12% (0.09–0.14)
Age-standardised prevalence of blindness in Women 50+ years	0.13% (0.10–0.15)	0.01% (0.01–0.01)	0.01% (0.01–0.01)	0.10% (0.08–0.12)	0.09% (0.07–0.12)	0.34% (0.27–0.42)	0.16% (0.12–0.19)	0.11% (0.09–0.14)
**Percentage of all blindness**	**8.60% (7.22**–**9.99)**	**2.07% (1.64**–**2.54)**	**2.66% (2.11**–**3.24)**	**5.96% (4.92**–**7.04)**	**6.15% (5.03**–**7.40)**	**12.71% (10.58**–**14.82)**	**9.34% (7.67**–**10.94)**	**5.00% (4.17**–**5.96)**
**Number of people with MSVI (thousands)**	**157,000 (140,000**–**176,000)**	**9660 (8560**–**10,900)**	**17,100 (15,200**–**18,900)**	**14,700 (13,000**–**16,300)**	**12,800 (11,400**–**14,400)**	**53,900 (47,800**–**60,900)**	**39,700 (35,400**–**44,600)**	**9620 (8480**–**10,900)**
Number of Men with MSVI (thousands)	73,300 (65,400–81,900)	3830 (3380–4310)	8240 (7300–9150)	6550 (5820–7300)	6380 (5660–7120)	25,600 (22,700–28,900)	18,200 (16,200–20,400)	4520 (3970–5120)
Number of Women with MSVI (thousands)	84,100 (74,900–93,900)	5830 (5180–6600)	8850 (7900–9810)	8140 (7180–9070)	6460 (5740–7240)	28,200 (25,000–31,900)	21,500 (19,200–24,200)	5100 (4500–5760)
**Number of people with MSVI aged 50+ years (thousands)**	**86,100 (74,200**–**101,000)**	**6340 (5400**–**7480)**	**8940 (7680**–**10,400)**	**5780 (4950**–**6780)**	**4680 (3960**–**5550)**	**32,150 (27,500**–**37,900)**	**25,050 (21,500**–**29,300)**	**3210 (2730**–**3800)**
Number of Men with MSVI aged 50+ years (thousands)	39,000 (33,600–45,810)	2270 (1920–2710)	3720 (3200–4310)	2610 (2240–3070)	2280 (1900–2690)	15,600 (13,300–18,400)	11,100 (9550–13,100)	1480 (1260–1750)
Number of Women with MSVI aged 50+ years (thousands)	47,100 (40,600–55,200)	4070 (3470–4780)	5220 (4490–6010)	3170 (2720–3710)	2400 (2030–2840)	16,600 (14,200–19,500)	13,900 (12,000–16,200)	1730 (1470–2050)
**Age-standardised prevalence of MSVI**	**1.91% (1.71**–**2.13)**	**1.85% (1.65**–**2.06)**	**1.37% (1.21**–**1.53)**	**2.39% (2.12**–**2.65)**	**2.24% (2.00**–**2.50)**	**3.37% (2.99**–**3.81)**	**1.60% (1.43**–**1.78)**	**1.24% (1.10**–**1.38)**
Age-standardised prevalence of MSVI: Men	1.83% (1.63–2.04)	1.69% (1.50–1.88)	1.41% (1.25–1.60)	2.22% (1.97–2.46)	2.17% (1.93–2.42)	3.25% (2.89–3.65)	1.49% (1.33–1.66)	1.21% (1.08–1.35)
Age-standardised prevalence of MSVI: Women	2.00% (1.78–2.23)	1.98% (1.76–2.22)	1.31% (1.16–1.47)	2.56% (2.27–2.86)	2.32% (2.07–2.59)	3.51% (3.11–3.97)	1.70% (1.52–1.90)	1.27% (1.12–1.42)
**Age-standardised prevalence of MSVI 50+ years**	**4.58% (3.96**–**5.37)**	**4.51% (3.85**–**5.31)**	**1.94% (1.67**–**2.25)**	**4.28% (3.68**–**5.00)**	**4.73% (4.02**–**5.54)**	**10.28% (8.82**–**12.06)**	**3.94% (3.39**–**4.56)**	**3.16% (2.73**–**3.70)**
Age-standardised prevalence of MSVI in Men 50+ years	4.41% (3.80–5.16)	3.97% (3.41–4.70)	1.80% (1.55–2.10)	4.21% (3.62–4.92)	4.57% (3.88–5.36)	10.19% (8.74–11.89)	3.66% (3.16–4.26)	3.13% (2.70–3.65)
Age-standardised prevalence of MSVI in Women 50+ years	4.75% (4.09–5.56)	4.89% (4.18–5.76)	2.05% (1.76–2.38)	4.35% (3.74–5.09)	4.88% (4.14–5.72)	10.40% (8.93–12.18)	4.19% (3.62–4.87)	3.19% (2.74–3.74)
**Percentage of all MSVI**	**53.39% (47.56**–**59.51)**	**53.69% (47.61**–**60.63)**	**55.00% (49.02**–**60.95)**	**60.00% (53.21**–**66.69)**	**58.76% (52.21**–**65.72)**	**55.99% (49.64**–**63.32)**	**47.84% (42.61**–**53.69)**	**47.06% (41.49**–**53.29)**

Data are presented for the whole population (bold), by sex breakdown and for those 50+ years. Figures in parentheses reflect 95% uncertainty intervals. Count data are presented to three significant figures, and percentages to two decimal places.

As a percentage of all types of blindness, the burden of blindness due to URE globally is 8.60% (95% UI 7.22–9.99%) and is greatest for the super regions of South Asia (12.71%, 95% UI 10.58–14.82%) and Southeast Asia, East Asia and Oceania (9.34%, 95% UI 7.67–10.94%). These updated data estimate that URE is the leading cause of MSVI globally, accounting for 53.39% (95% UI 47.56–59.51%) of all cases. Focusing on blindness due to URE in those aged 50+ years, South Asia accounts for the largest age-standardised prevalence (0.33% (95% UI 0.26–0.40%)), followed by the super-regions of Southeast Asia, East Asia and Oceania (0.15% (95% UI 0.12–0.18%)) and Sub-Saharan Africa (0.11% (95% UI 0.09–0.14%)).

**Fig. 1:**
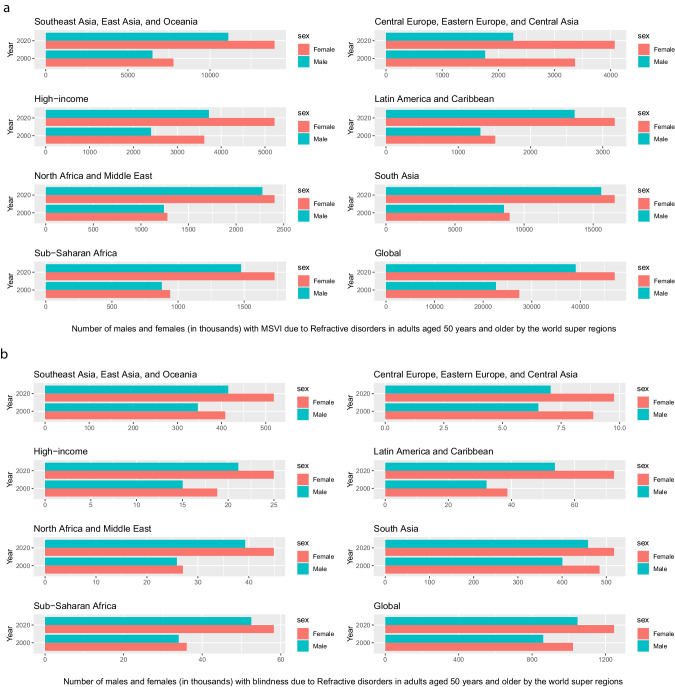
Bar charts demonstrating the number of men and women with blindness and MSVI due to URE in 2000 and 2020 by seven World GBD super-regions and globally. **a** indicates numbers for MSVI and **b** blindness. Note that scales (values should be multiplied by 1000) are not the same between charts, but rather serve to highlight the differences across the time period and sex differences within GBD super-regions.

**Fig. 2:**
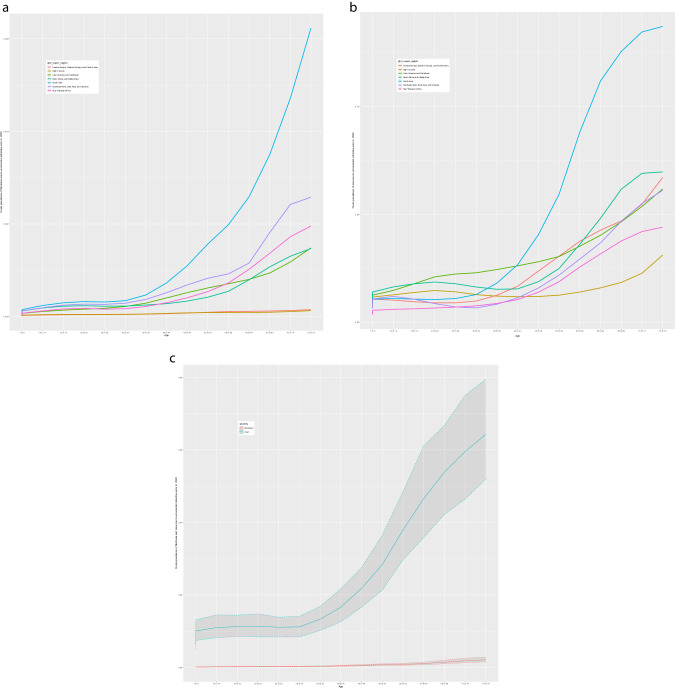
Crude prevalence of blindness and MSVI due to URE by region and globally by age. **a** Crude prevalence of blindness due to URE in 2020 by seven World GBD super regions by age. **b** Crude prevalence of MSVI due to URE in 2020 by seven World GBD super regions by age. **c** Crude prevalence of Blindness (red) and MSVI (cyan) due to URE in 2020 globally by age, with 95% UI indicated as shading.

The overall age-standardised prevalence of blindness due to URE in those aged 50+ years was 0.12% (95% UI 0.10–0.15%), and 4.58% for MSVI (95% UI 3.96–5.37%). Figure 1 shows the number of men and women aged 50+ years with blindness and MSVI due to URE in 2020 in the seven World GBD super regions, and includes the global total for overall comparison. Figure 2 presents the crude prevalence of blindness and MSVI due to URE in 2020 across super regions.

Table 2 presents the percentage change in crude prevalence of MSVI and blindness due to URE in men and women aged 50 years and older between 2000 and 2020 for the seven World GBD super regions (see Appendix 1 for all 21 GBD World regions). Over this time period the number of cases of blindness and MSVI increased by +21.8% and +72.0%% respectively, with the greatest increase in the Latin America and Caribbean super-region for both blindness and MSVI. However, the age-standardised prevalence of URE blindness in those 50+ years decreased significantly, by −30.5% (95% UI −30.7 to −30.3) during this time period. The global age-standardised prevalence of MSVI due to URE in those aged 50+ years modestly decreased by −2.4% (95% UI −2.6 to −2.2%) between 2000 and 2020, but with some regional variations. The Latin America and Caribbean super-region demonstrated a slight increase in age-standardised prevalence of MSVI due to URE of +0.8% (95% UI + 0.7 to +1.0%), and the High-Income super-region had no change (+0.1%, 95% UI −0.1 to +0.3).

**Table 2:** Percentage change in crude prevalence, case number and age-standardised prevalence of MSVI and blindness due to URE in adults aged 50 years and older in the 7 Super Regions between 2000 and 2020.

	MSVI caused by URE									Blindness caused by URE								
	Percentage change in Crude Prevalence between 2000 and 2020			Percentage Change in Number of Cases between 2000 and 2020			Percentage Change in Age-standardised prevalence between 2000 and 2020			Percentage Change in Crude Prevalence between 2000 and 2020			Percentage Change in Number of Cases between 2000 and 2020			Percentage Change in Age-standardised prevalence between 2000 and 2020		
Region	Men	Women	**Both**	Men	Women	**Both**	Men	Women	**Both**	Men	Women	**Both**	Men	Women	**Both**	Men	Women	**Both**
**GLOBAL**	−2.2% (−2.4 to −2.0)	−2.3% (−2.5 to−2.1)	**−2.3% (−2.5 to −2.1)**	+72.5% (72.2 to 72.8)	+71.6% (71.3 to 71.9)	**+72.0% (71.7 to 72.3)**	−3.0% (−3.1 to −2.8)	−2.0% (−2.2 to −1.8)	**−2.4% (−2.6 to −2.2)**	−31.0% (−31.2 to −30.8)	−30.6% (−30.8 to −30.5)	**−30.8% (−31.0 to −30.6)**	+21.7% (21.4 to 22.1)	+21.9% (21.6 to 22.2)	**+21.8% (21.5 to 22.2)**	−31.6% (−31.8 to −31.4)	−29.9% (−30.0 to −29.7)	**−30.5% (−30.7 to −30.3)**
**Central Europe, Eastern Europe & Central Asia**	−1.1% (−1.3 to −0.9)	−2.2% (−2.4 to −2.0)	**−2.1% (−2.3 to −1.9)**	+28.3% (28.1 to 28.6)	+21.0% (20.7 to 21.2)	**+23.5% (23.3 to 23.7)**	−2.3% (−2.5 to −2.1)	−2.3% (−2.5 to −2.2)	**−2.8% (−2.9 to −2.6)**	−16.8% (−17.0 to −16.5)	−11.1% (−11.4 to −10.8)	**−13.5% (−13.8 to −13.2)**	+8.0% (7.6 to 8.3)	+10.0% (9.7 to 10.4)	**+9.2% (8.8 to 9.5)**	−17.4% (−17.8 to −17.5)	−10.7% (−10.4 to −11.1)	**−13.8% (−14.1 to −14.4)**
**High Income**	+5.3% (5.1 to 5.5)	+3.6% (3.4 to 3.8)	**+4.0% (3.8 to 4.2)**	+55.0% (54.7 to 55.2)	+44.3% (44.1 to 44.6)	**+48.6% (48.3 to 48.8)**	−0.1% (−0.3 to 0.0)	+1.0% (0.8 to 1.2)	**+0.1% (−0.1 to 0.3)**	−4.6% (−4.9 to −4.3)	−4.6%(−4.9 to −4.4)	**−4.7%(−4.9 to −4.4)**	+40.4% (40.0 to 40.8)	+32.9% (32.5 to 33.2)	**+36.2% (35.8 to 36.6)**	−7.9%(−8.2 to −7.7)	−6.9%(−7.1 to −6.8)	**−7.2%(−7.5 to −7.0)**
**Latin America and Caribbean**	+1.4% (1.2 to 1.6)	+2.3% (2.1 to 2.5)	**+1.9% (1.8 to 2.1)**	+99.8% (99.4 to 100.2)	+109.4% (109.1 to 109.8)	**+105.0% (104.6 to 105.4)**	+0.4% (0.2 to 0.5)	+1.2% (1.0 to 1.4)	**+0.8% (0.7 to 1.0)**	−15.0% (−15.2 to −14.8)	−8.5% (−8.7 to −8.3)	**−11.3% (−11.5 to −11.1)**	+67.5% (67.1 to 68.0)	+87.3% (86.8 to 87.8)	**+78.3% (77.9 to 78.8)**	−15.9% (−16.1 to −15.7)	−10.4% (−10.6 to −10.1)	**−12.7% (−12.9 to −12.5)**
**North Africa and Middle East**	−11.8% (−12.0 to −11.6)	−9.6% (−9.8 to −9.4)	**−10.7% (−10.9 to −10.5)**	+83.2% (82.9 to 83.6)	+88.1% (87.8 to 88.5)	**+85.7% (85.4 to 86.1)**	−9.0% (−9.2 to −8.8)	−8.1% (−8.3 to −7.9)	**−8.5% (−8.7 to −8.3)**	−27.0% (−27.2 to −26.8)	−20.2% (−20.5 to −20.0)	**−23.5% (−23.8 to −23.3)**	+51.7% (51.3 to 52.2)	+66.0% (65.5 to 66.4)	**+59.0% (58.5 to 59.5)**	−23.2% (−23.4 to −23.0)	−18.3% (−18.5 to −18.1)	**−20.7% (−20.9 to −20.5)**
**South Asia**	−3.3% (−3.5 to −3.2)	−8.5% (−8.7 to −8.3)	**−5.9% (−6.1 to −5.7)**	+82.4% (82.1 to 82.8)	+85.0% (84.7 to 85.4)	**+83.8% (83.4 to 84.1)**	−4.3% (−4.5 to −4.1)	−8.9% (−9.1 to −8.8)	**−6.7% (−6.8 to −6.5)**	−39.4% (−39.5 to −39.2)	−47.2% (−47.4 to −47.1)	**−43.6% (−43.7 to −43.4)**	+14.5% (14.2 to 14.8)	+6.7% (6.4 to 7.0)	**+10.2% (9.9 to 10.5)**	−42.1% (−42.2 to −41.9)	−50.0% (−50.2 to −49.9)	**−46.3% (−46.5 to −46.2)**
**Southeast Asia, East Asia and Oceania**	−13.5% (−13.7 to −13.4)	−12.2% (−12.3 to −12.0)	**−12.7% (−12.8 to −12.5)**	+71.0% (70.7 to 71.3)	+79.2% (78.9 to 79.5)	**+75.5% (75.1 to 75.8)**	−14.9% (−15.1 to −14.8)	−12.3% (−12.5 to −12.2)	**−13.5% (−13.7 to −13.4)**	−39.4% (−39.5 to −39.2)	−37.8% (−37.9 to −37.6)	**−38.4% (−38.6 to −38.3)**	+19.9% (19.6 to 20.2)	+27.0% (26.6 to 27.3)	**+23.7% (23.4 to 24.0)**	−39.8% (−39.9 to −39.6)	−37.6% (−37.8 to −37.5)	**−38.8% (−38.9 to −38.6)**
**Sub−Saharan Africa**	−4.9% (−5.1 to −4.7)	−5.2% (−5.3 to −5.0)	**−4.9% (−5.1 to −4.7)**	+68.3% (68.0 to 68.6)	+84.0% (83.6 to 84.4)	**+76.4% (76.1 to 76.8)**	−3.7% (−3.8 to −3.5)	−3.3% (−3.5 to −3.1)	**−3.4% (−3.5 to −3.2)**	−12.8% (−13.0 to −12.6)	−16.7% (−17.0 to −16.5)	**−14.8% (−15.1 to −14.6)**	+54.3% (53.8 to 54.7)	+61.5% (61.1 to 62.0)	**+58.0% (57.6 to 58.4)**	−10.6% (−10.8 to −10.4)	−14.0% (−14.2 to −13.7)	**−12.5% (−12.7 to −12.2)**

Percentage change to 1 decimal place and figures in parentheses reflect 95% uncertainty intervals. Data in bold indicate totals for both sexes.

By a clear margin, South Asia had the highest regional 50+ years age-standardised URE blindness and MSVI prevalence in 2020 (blind: 0.3%, 95% UI 0.3–0.4; MSVI: 10.3%; 95% UI 8.8–12.1%) (Table 1), but also demonstrated the greatest reductions in age-standardised URE blindness between 2000 and 2020 (−46.3% (95% UI −46.5 to −46.2%)) (Table 2).

Globally, the age-standardized ratio of women to men for URE blindness was 1.05:1.00 in 2020 and 1.03:1.00 in 2000. For MSVI, this ratio was 1.08:1.00 in 2020 and 1.06:1.00 in 2000. Thus, in 2020, women continue to suffer an excess burden, with the age-standardized prevalence of women exceeding that of men by 4.76% for URE blindness and 7.40% for URE MSVI. Men exhibited a greater 20-year reduction in age-standardised prevalence compared to women for both blindness and MSVI: MSVI −3.0% (95% UI −3.1 to −2.8) in men, −2.0% (95% UI −2.2 to −1.8) in women; blindness −31.6% (95% UI −31.8 to −31.4) in men, −29.9% (95% UI −30.0 to −29.7) in women. Regionally, women have made smaller gains than men in the reduction of age−standardised prevalence of MSVI due to URE, particularly in the super regions of Central Europe, Eastern Europe and Central Asia, North Africa and Middle East, and Latin America and Caribbean. In the High-Income super-region, age-standardised prevalence of blindness has actually increased modestly for woman at +1.0% (95% UI 0.8 to 1.2%) compared to men −0.1% (95% UI −0.3 to 0.0). However, it is notable that in South Asia, there has been a greater reduction in age-standardised prevalence of both blindness and MSVI for women compared to men (percentage reduction blindness; women −50.0% (95% UI −50.2 to −49.9), men −42.1% (95% UI −42.2 to −41.9): percentage reduction MSVI; women −8.9% (95% UI −9.1 to −8.8), men −4.3% (95% UI −4.5 to −4.1)), although the burden remains substantial.

Table 3 presents the number of people, men and women with near VI from uncorrected presbyopia in the seven super regions. In 2020, an estimated 419 million (95% UI 295–562 million) people aged 50+ had near VI from uncorrected presbyopia globally, with an age-standardised prevalence of 22.3% (95% UI 15.8–29.9%). Approximately 70% of global near VI from presbyopia occurred in two super regions: South Asia and Southeast Asia, East Asia and Oceania (293 million).

**Table 3:** Number and age-standardised prevalence of people with uncorrected presbyopia aged 50+ years (>N6/N8 at 40 cm when best-corrected distance visual acuity was 6/12 or better) in 7 Super Regions in 2020.

	GLOBAL	Central Europe, Eastern Europe, and Central Asia	High income	Latin America and Caribbean	North Africa and Middle East	South Asia	Southeast Asia, East Asia, and Oceania	Sub-Saharan Africa
Total population 2020 (thousands)	7,890,000	417,000	1,090,000	602,000	632,000	1,840,000	2,190,000	1,110,000
**Number of people with uncorrected presbyopia (aged 50+ years) (thousands)**	**419,000 (295,000**–**562,000)**	**39,800 (28,300**–**53,500)**	**11,400 (7700**–**15,900)**	**24,910 (17,600**–**33,600)**	**12,200 (8410**–**16,800)**	**124,000 (86,600**–**166,000)**	**169,000 (118,000**–**227,000)**	**37,800 (27,200**–**49,700)**
Number of Men with uncorrected presbyopia (thousands)	186,000 (130,000–251,000)	14,500 (10,200–19,600)	5020 (3400–6970)	11,200 (7820–15,100)	5800 (3900–8020)	57,900 (40,500–78,300)	75,000 (52,000 to 102,000)	17,000 (12,200–22,400)
Number of Women with uncorrected presbyopia (thousands)	233,000 (164,000– 311,000)	25,300 (18,200–33,900)	6350 (4300–8900)	13,700 (9700–18,500)	6440 (4470–8770)	66,300 (46,310 to 88,100)	93,700 (66,000–125,000)	20,700 (15,000 to 27,200)
**Age-standardised prevalence of uncorrected presbyopia**	**22.33% (15.81**–**29.91)**	**27.81% (19.89**–**37.41)**	**2.37% (1.59**–**3.31)**	**18.85% (13.33**–**25.38)**	**13.18% (9.23**–**17.89)**	**38.89% (28.30**–**53.17)**	**26.63% (18.81**–**35.78)**	**37.54% (27.33**–**49.08)**
Age-standardised prevalence of uncorrected presbyopia: Men	21.11% (14.92–28.29)	25.89% (18.51–34.58)	2.35% (1.57–3.28)	18.66% (13.19–25.16)	12.60% (8.72–17.27)	37.78% (26.71–50.35)	24.71% (17.51–33.29)	36.30% (26.31–47.28)
Age-standardised prevalence of uncorrected presbyopia: Women	23.43% (16.52–31.36)	29.10% (20.93–39.08)	2.38% (1.60–3.33)	19.01% (13.44–25.69)	13.74% (9.66–18.68)	41.93% (29.63–55.68)	28.36% (19.94–38.00)	38.62% (28.12–50.22)

Data in parentheses are 95% uncertainty intervals. Count data are presented to three significant figures, and percentages to two decimal places. Data in bold indicate totals for both sexes.

**Fig. 3:**
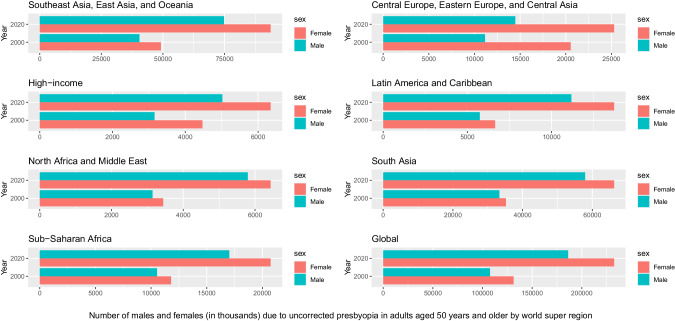
Comparison of the number of men and women with near vision impairment due to uncorrected presbyopia in 2000 and 2020 by seven World GBD super regions, with Global total bottom right panel. Note scales (values should be multiplied by 1000) are not the same between charts, but rather serve to highlight the differences across the time period and sex differences within GBD super regions.

Table 4 presents the percentage change in crude prevalence of near VI due to uncorrected presbyopia in men and women aged 50 years and older between 2000 and 2020. Over this time period, the number of cases of near VI due to presbyopia increased substantially (+75.3% (95% UI + 74.6 to +76.0)), while the crude prevalence demonstrated a modest reduction for men (−1.8% (95% UI −2.2 to −1.4%)), but an increase of +0.8% (95% UI 0.4–1.2%) for women. Figure 3 further illustrates these sex differences across super regions, demonstrating significant increases in the number of cases in the 20-year period, with a disproportionate increase for women. The number of cases of near VI due to presbyopia increased in all super regions, ranging from 25.5% (95% UI + 25.0 to +25.9%) in Central Europe, Eastern Europe, and Central Asia to 101% (95% UI + 100.2 to +101.7%) in Latin America and Caribbean super-region. However, the percentage change in crude prevalence decreased in all super regions over the 20-year period except for the High-Income super-region which had a +4.3% (95% UI + 3.9 to +4.7) increase.

**Table 4:** Percentage change in crude prevalence and case number of uncorrected presbyopia in adults aged 50 years and older in the 7 Super Regions between 2000 and 2020.

	Near VI caused by uncorrected presbyopia					
	Percentage Change in Crude Prevalence between 2000 and 2020			Percentage Change in Number of Cases between 2000 and 2020		
Region	Men	Women	Both	Men	Women	Both
**GLOBAL**	−1.8% (−2.2 to −1.4)	+0.8% (+0.4 to +1.2)	−0.4% (−0.8 to 0.0)	+73.2% (+72.5 to +73.8)	+77.1% (+76.4 to +77.7)	+75.3% (+74.6 to +76.0)
**Central Europe, Eastern Europe & Central Asia**	0.0% (−0.4 to +0.4)	−0.5% (−0.9 to −0.1)	−0.6% (−0.9 to −0.2)	+29.8% (+29.3 to +30.3)	+23.1% (+22.7 to +23.6)	+25.5% (+25.0 to +25.9)
**High Income**	+8.1% (+7.6 to +8.5)	+1.9% (+1.5 to +2.3)	+4.3% (+3.9 to +4.7)	+59.0% (+58.3 to +59.7)	+41.9% (+41.3 to +42.5)	+49.0% (+48.4 to +49.6)
**Latin America and Caribbean**	−1.2% (−1.6 to −0.8)	+0.8% (+0.4 to +1.2)	−0.1% (−0.4 to 0.3)	+94.8% (+94.0 to +95.5)	+106.3% (+105.5 to +107.1)	+101.0% (+100.2 to +101.7)
**North Africa and Middle East**	−11.2% (−11.6 to −10.9)	−10.1% (−10.5 to −9.8)	−10.7% (−11.0 to −10.3)	+84.5% (+83.7 to +85.2)	+87.0% (+86.2 to +87.7)	+85.8% (+85.0 to +86.6)
**South Asia**	−8.0% (−8.3 to −7.6)	−7.0% (−7.3 to −6.6)	−7.3% (−7.6 to −6.9)	+73.7% (+73.1 to +74.4)	+88.1% (+87.4 to +88.8)	+81.1% (+80.4 to +81.8)
**Southeast Asia, East Asia and Oceania**	−6.5% (−6.9 to −6.2)	−6.6% (−6.9 to −6.2)	−6.4% (−6.8 to −6.1)	+84.8% (+84.1 to +85.6)	+90.6% (+89.8 to +91.3)	+88.0% (+87.3 to +88.7)
**Sub-Saharan Africa**	−8.7% (−9.0 to −8.4)	−9.4% (−9.7 to −9.0)	−8.9% (−9.2 to −8.5)	+61.5% (+61.0 to +62.1)	+75.9% (+75.2 to +76.5)	+69.1% (+68.5 to +69.7)

Percentage change to 1 decimal place and figures in parentheses reflect 95% uncertainty intervals.

## Discussion

This study provides up-to-date global and regional, sex-specific and age-specific estimates and temporal trends for vision impairment due to URE, both for distance and near vision impairment. Our study reveals that URE remains a leading cause of MSVI, affecting 157 million individuals worldwide in 2020, and MSVI due to URE accounts for 57% of all MSVI globally. Notably, although there is some variation across the super regions, the percentage of MSVI due to URE remains above 47% in all areas, underscoring the persistent and substantial global burden of avoidable vision loss caused by URE.

In the 20-year period up to 2020, VISION 2020: the Right to Sight initiative sought to prevent avoidable sight loss, and the subsequent Global Action Plan adopted by the WHA in 2013 set a target for a 25% reduction in the prevalence of avoidable vision impairment by 2019 from the baseline of 2010. While progress in reducing the global burden has been made, this target was not achieved [29], highlighting the need for continued focus and effort to eliminate avoidable sight loss.

Encouragingly, the age-standardised prevalence of blindness due to URE in those aged 50+ years has decreased substantially from 2000 to 2020, potentially reflecting the targeted efforts countries have adopted to tackle severe sight loss. This may in part be explained by the increased use of intra-ocular lenses in cataract surgery over the last 20–30 years, leading to a reduction in blindness due to aphakia [38]. In contrast, the age-standardised prevalence of MSVI due to URE in those aged 50+ years only decreased modestly between 2000 and 2020.

The reductions we observed in age-standardised prevalence are counterbalanced by a striking increase in the unadjusted burden of blindness and MSVI due to URE, meaning that the total number of affected persons in the world has risen. This is driven by two key factors: continued global population growth, which is estimated to reach 10.4 billion in 2100, and an ageing population [39]. In common with the majority of vision-impairing ocular diseases, the likelihood of MSVI and blindness due to URE rapidly increases with age, as shown in Fig. 2. UN projections report that between 2020 and 2050 the global population of those aged 65+ years is expected to double from 703 million to 1.5 billion, and that by 2050, one in six people in the world will be aged ≥ 65 years [40].

The super-region of South Asia, comprising countries including India and Pakistan, had the greatest burdens of blindness and MSVI, with a disproportionately high prevalence of older age groups. While globally there was a reduction in age-standardised prevalence of MSVI due to URE, the super-region of Latin America and Caribbean actually demonstrated an increase of +0.8% (95% UI 0.4–1.3). These regional differences in the prevalence of vision loss are likely due to variations in availability of affordable refractive services, particularly in rural locations, and in social conditions. This is evidenced by a recent study investigating the eREC across regions, which demonstrated substantial differences in eREC between super regions in 2021, with 79.1% coverage in the High-Income super-region (95% CI 72·4–85·0), compared to 6.7% in Sub-Saharan Africa (95% CI 3·1–9·0) and 9.0% in South Asia (95% CI 6·5–12·0%) [41].

New eyecare service development has not kept pace with the increasing population demands in any region of the world, and continued population ageing will further increase existing burdens. Alleviating this shortfall will require a combination of capacity-building of trained eyecare personnel, expansion of community-based screening services for diagnosis of refractive errors, development of infrastructure for spectacle provision, outreach efforts to drive demand, and novel technical approaches to allow more services to be delivered by available, less fully-trained cadres. The WHO World Report on Vision, 2019 [17] sets out four key areas to increase access to eyecare services: (i) Increase of the availability of services through training and improved infrastructure; (ii) Increase the accessibility of services to those who need them; (iii) Increase the affordability of services, and (iv) Increase of the acceptability of refractive services, through awareness raising.

While the burden of vision impairment increases with age, focusing only on the population aged 50 years and above provides an incomplete view of vision impairment due to URE, which also frequently affects younger persons. While we report that those aged 50+ years with MSVI total 86 million in 2020, this only accounts for 55% of all MSVI (167 million). For younger people, the burden of URE is likely driven by the concerning global increase in myopia [42], with recent evidence showing these trends are not only confined to Asian populations [43]. However, there remains a paucity of data on vision impairment due to URE in children and younger adults, which needs to be redressed.

A disproportionate number of women continue to be affected by vision impairment due to URE. This is observed globally and across the majority of super regions. Interestingly for presbyopia, while the global crude prevalence decreased for men by −1.8% (−2.2 to −1.4%) from 2000 to 2020, there was an increase for women of +0.8% (+0.4 to +1.2%). It is important to emphasise that these differences persist after age adjustment, and are not simply an artefact of women living longer. This unfair burden among women is likely driven by cultural and social inequities, with less financial autonomy, male prioritisation, and child- and home-care responsibilities [44]. This persistent gap must be addressed through targeted strategies to increase their access to refractive care.

The burden of near visual impairment due to uncorrected presbyopia is another critical area of concern highlighted by our study, with nearly 420 million people aged 50+ years affected by uncorrected presbyopia. There are huge disparities in the age-standardised prevalence of uncorrected presbyopia across super regions, with for example, only a 2.4% prevalence in the High-Income super-region compared with 38.9% in South Asia. This finding is supported by a systematic review reporting the greatest burden of presbyopia in rural areas in low-resource countries [45]. Looking at temporal data, some super regions demonstrated a reduction in crude prevalence but in all areas the number of cases increased significantly, likely due to the ageing population globally and also improvements in data availability in the last 20 years. It was not possible to generate age-standardised estimates due to sparsity of data. The combination of high, rapidly rising burden and the paucity of data underscores the need for more attention to presbyopia among both researchers and health service planners.

The large burden of uncorrected presbyopia may in part reflect a view that correction for near VI is somehow less important than for distance VI, but studies have shown that vision impairment from URE affects the quality of life to a similar degree whether at distance or near VI [14]. Furthermore, a recent study [46] reported on the considerable productivity loss from un- and under-corrected presbyopia in LICs and LMICs. Using GBD data, the authors estimated 238 million people of working age (15–65 years) in LMICs had uncorrected presbyopia, and estimated the resulting direct productivity loss at $54 billion dollars, using productivity-adjusted-life-years. The potential for presbyopic correction to improve real-world work productivity is underscored by recent trials [2].

The strengths of this updated review and data analysis up to 2020 include the addition of new data sources, particularly more RAAB surveys, which enable improvements in disaggregation by cause and a wider coverage of geographical regions in our analysis. This is also the first time we combine reports on the impact of distance and near visual impairment due to URE and presbyopia. However, there remain several LICs and LMICs in regions such as central sub-Saharan Africa, Central Asia, and central and eastern Europe, with scant population-based data where estimates rely on extrapolation from other regions. While our modelling has controlled for a range of confounding factors, it is possible that blindness and MSVI due to URE are underreported. Furthermore, due to data sparsity, we did not include mild visual impairment in this dataset but used a definition of <6/18 for MSVI, so again these data underreport the potential burden of distance vision impairment compared to other studies. Finally, it is possible that the trajectory of the prevalence of vision impairment due to URE might be altered owing to the COVID-19 global pandemic, with reports emerging of an increase in the prevalence of myopia attributed to changes in lifestyle during the pandemic [47]. Future directions for research and policy should be develop population screening services, accurate reporting mechanisms and registries to effectively measure the burden of avoidable vision impairment due to URE, to strengthen data from younger populations, and focus efforts on developing refractive services in LICs and LMICs to fill the data gaps to achieve greater geographical coverage.

## Conclusions

Data from the last 20 years show that the absolute number of people with URE is rising due to population growth and ageing. URE remains a leading global cause of MSVI among persons aged 50+ years, affecting 86 million individuals and accounting for 53.4% of the total figure. This, coupled with the huge burden of near vision impairment due to uncorrected presbyopia, highlights the urgent need for novel and fresh approaches to refractive service delivery. While progress has been made in the last two decades, a reduction in the burden of vision impairment from URE can be realised by adding refractive services to universal health coverage and otherwise improving availability of, and access to, spectacle provision. Though the need is greater in some global regions, URE has not been fully addressed anywhere, and the resulting productivity losses and reduction in quality of life should not be overlooked for any country. Over this decade, the target set by the 73rd WHA member states in 2021, a 40% increase in eREC by 2030, will provide critical leverage to accelerate our efforts to tackle avoidable blindness due to URE.

URE remains the leading cause of MSVI, though spectacle provision is the simplest and least invasive treatment available for any ocular condition. This is a source of frustration after decades of work on VISION 2020, but it underscores the opportunity to accelerate progress towards what is arguably the most attainable goal in vision care, that of eliminating URE.

## Summary

### What was known before

- Uncorrected refractive error (URE) is the leading cause of vision impairment globally among both adults and children, and contributes to reduced educational and economic opportunities, decreased quality of life and an increased burden of mortality
- Visual impairment is a significant global health concern, and the ‘World Report on Vision’ by the World Health Organisation in 2019 called for the routine measurement of refractive error services coverage as a means to address the UN Sustainable Development Goal 3.8 of universal health coverage
- Uncorrected refractive error (URE) is readily treated with spectacles, making it one of the most cost-effective healthcare interventions, both for distance visual impairment and near visual impairment due to presbyopia
- The need for new population data on vision impairment is vital to monitor and measure success against global targets to increase the coverage of refractive error services by 40% by 2030

### What this study adds

- This study provides up-to-date global and regional, sex-specific and age-specific estimates and temporal trends for vision impairment due to uncorrected refractive error, both for distance and near vision impairment.
- We examined age-adjusted and sex-adjusted differences in the contribution of uncorrected refractive error to vision impairment, with a focus on older age groups
- We incorporated studies from an updated systematic review for a total of 243 sources from 73 countries
- Our study reveals that over the last 20 years, the absolute number of people with URE has risen due to population growth and ageing, with a continued disproportionate burden by region and sex
- Uncorrected refractive error (URE) remains a leading cause of MSVI, affecting 157 million individuals worldwide in 2020, and MSVI due to URE accounts for 57% of all MSVI globally
- Furthermore, an estimated 419 million people aged 50+ had near VI from uncorrected presbyopia globally in 2020
- These data underscore the persistent and substantial global burden of avoidable vision loss caused by uncorrected refractive error, highlighting the urgent need for novel and fresh approaches to refractive service delivery.

### Supplementary information

Appendix 1

Appendix 2 - Contributions by Authors

## Data Availability

The data that support the findings of this study are not openly available due to reasons of sensitivity and are available from the coordinator of the Vision Loss Expert Group (Professor Rupert Bourne; rb@rupertbourne.co.uk) upon reasonable request. Data are located in controlled access data storage at Anglia Ruskin University.

## References

[1] Ma X, Zhou Z, Yi H, Pang X, Shi Y, Chen Q, et al. Effect of providing free glasses on children’s educational outcomes in China: cluster randomized controlled trial. BMJ. 2014;349:23. 10.1136/bmj.g5740 PMC4172821 25249453

[2] Reddy PA, Congdon N, MacKenzie G, Gogate P, Wen Q, Jan C, et al. Effect of providing near glasses on productivity among rural Indian tea workers with presbyopia (PROSPER): a randomised trial. Lancet Glob Health. 2018;6:e1019–e1027. 30049615 10.1016/S2214-109X(18)30329-2

[3] Slavin RE, Collins ME, Repka MX, Friedman DS, Mudie LI, Owoeye JO, et al. In plain sight: reading outcomes of providing eyeglasses to disadvantaged children. J Educ Stud Placed Risk. 2018;23:250–8. 10.1080/10824669.2018.1477602

[4] Naidoo KS, Fricke TR, Frick KD, Jong M, Naduvilath TJ, Resnikoff S, et al. Potential lost productivity resulting from the global burden of myopia: systematic review, meta-analysis, and modelling. Ophthalmology. 2019;126:338–46. 30342076 10.1016/j.ophtha.2018.10.029

[5] Nie JC, Pang XP, Wang L, Rozelle S, Sylvia S. Seeing is believing: experimental evidence on the impact of eyeglasses on academic performance, aspirations, and dropout among junior high school students in rural China. Econ Dev Cult Change. 2020;68:335–55. 10.1086/700631

[6] Marques AP, Ramke J, Cairns J, Butt T, Zhang JH, Muirhead D, et al. Global economic productivity losses from vision impairment and blindness. E Clin. Med. 2021;35:26. 10.1016/j.eclinm.2021.100852 PMC8093883 33997744

[7] Ramrattan RS, Wolfs RC, Panda-Jonas S, Jonas JB, Bakker D, Pols HA, et al. Prevalence and causes of visual field loss in the elderly and associations with impairment in daily functioning: the Rotterdam Study. Arch Ophthalmol. 2001;119:1788–94. 11735788 10.1001/archopht.119.12.1788

[8] McCarty CA, Nanjan MB, Taylor HR. Vision impairment predicts 5 year mortality. Br J Ophthalmol. 2001;85:322–6. 11222339 10.1136/bjo.85.3.322 PMC1723877

[9] Lee DJ, Gómez-Marín O, Lam BL, Zheng DD. Visual acuity impairment and mortality in US adults. Arch Ophthalmol. 2002;120:1544–50. 12427070 10.1001/archopht.120.11.1544

[10] Ehrlich JR, Ramke J, Macleod D, Burn H, Lee CN, Zhang JH, et al. Association between vision impairment and mortality: a systematic review and meta-analysis. Lancet Glob Health. 2021;9:e418–e430. 33607015 10.1016/S2214-109X(20)30549-0 PMC7966688

[11] Burton MJ, Ramke J, Marques AP, Bourne RRA, Congdon N, Jones I, et al. The Lancet Global Health Commission on Global Eye Health: Vision Beyond 2020. Lancet Glob Health. 2021;9:e489–e551. 33607016 10.1016/S2214-109X(20)30488-5 PMC7966694

[12] Baltussen R, Naus J, Limburg H. Cost-effectiveness of screening and correcting refractive errors in school children in Africa, Asia, America and Europe. Health Policy. 2009;89:201–15. 18621429 10.1016/j.healthpol.2008.06.003

[13] Frick KD, Riva-Clement L, Shankar MB. Screening for refractive error and fitting with spectacles in rural and urban India: cost-effectiveness. Ophthalmic Epidemiol. 2009;16:378–87. 19995203 10.3109/09286580903312277

[14] Fricke TR, Holden BA, Wilson DA, Schlenther G, Naidoo KS, Resnikoff S, et al. Global cost of correcting vision impairment from uncorrected refractive error. Bull World Health Organ. 2012;90:728–38. 23109740 10.2471/BLT.12.104034 PMC3471057

[15] Tahhan N, Papas E, Fricke TR, Frick KD, Holden BA. Utility and uncorrected refractive error. Ophthalmology. 2013;120:1736–44. 23664469 10.1016/j.ophtha.2013.02.014

[16] WHO. World Health Organization; Geneva: 2013. Universal eye health: a global action plan 2014–2019. https://www.who.int/publications/i/item/universal-eye-health-a-global-action-plan-2014-2019 Accessed 13 Feb 2023.

[17] World Health Organisation. World report on vision. Geneva; 2019. https://www.who.int/publications/i/item/9789241516570 Accessed 13 Feb 2023.

[18] United Nations. Sustainable Development Goals. 2015. https://sdgs.un.org/goals Accessed 14 May 2023.

[19] Hashemi H, Fotouhi A, Yekta A, Pakzad R, Ostadimoghaddam H, Khabazkhoob M. Global and regional estimates of prevalence of refractive errors: Systematic review and meta-analysis. J Curr Ophthalmol. 2017;30:3–22. 29564404 10.1016/j.joco.2017.08.009 PMC5859285

[20] Sun HP, Li A, Xu Y, Pan CW. Secular trends of reduced visual acuity from 1985 to 2010 and disease burden projection for 2020 and 2030 among primary and secondary school students in China. JAMA Ophthalmol. 2015;133:262–8. 25429523 10.1001/jamaophthalmol.2014.4899

[21] Lee JH, Jee D, Kwon JW, Lee WK. Prevalence and risk factors for myopia in a rural Korean population. Invest Ophthalmol Vis Sci. 2013;54:5466–71. 23838769 10.1167/iovs.13-12478

[22] He MG, Zeng JW, Liu YZ, Xu JJ, Pokharel GP, Ellwein LB. Refractive error and visual impairment in urban children in southern China. Invest Ophth Vis Sci. 2004;45:793–9. 10.1167/iovs.03-1051 14985292

[23] Walline JJ, Lindsley KB, Vedula SS, Cotter SA, Mutti DO, Ng SM, et al. Interventions to slow progression of myopia in children. Cochrane Database Syst Rev. 2020;1:13. 10.1002/14651858.CD004916.pub4 PMC6984636 31930781

[24] Mountjoy E, Davies NM, Plotnikov D, Smith GD, Rodriguez S, Williams CE, et al. Education and myopia: assessing the direction of causality by Mendelian randomisation. BMJ. 2018;361:6. 10.1136/bmj.k2022 PMC5987847 29875094

[25] Marmamula S, Narsaiah S, Shekhar K, Khanna RC, Rao GN. Visual Impairment in the South Indian State of Andhra Pradesh: Andhra Pradesh - Rapid Assessment of Visual Impairment (AP-RAVI) Project. PLoS ONE. 2013;8:e70120. 23894601 10.1371/journal.pone.0070120 PMC3720942

[26] Ahmed M, Shefali MK, Husain L, Khondaker M, Alauddin M, Hossain MA, et al. Vision impairment and productivity among female garment workers in Bangladesh: a cohort study. Asia Pac J Ophthalmol. 2022;11:79–84. 10.1097/APO.0000000000000485 35030134

[27] Bourne RRA, Flaxman SR, Braithwaite T. Magnitude, temporal trends, and projections of the global prevalence of blindness and distance and near vision impairment: a systematic review and meta-analysis. Lancet Glob Health. 2017;5:e888–e897. 28779882 10.1016/S2214-109X(17)30293-0

[28] Flaxman SR, Bourne RRA, Resnikoff S. Global causes of blindness and distance vision impairment 1990-2020: a systematic review and meta-analysis. Lancet Glob Health. 2017;5:e1221–e1234. 29032195 10.1016/S2214-109X(17)30393-5

[29] GBD 2019 Blindness and Vision Impairment Collaborators; Vision Loss Expert Group of the Global Burden of Disease Study. Causes of blindness and vision impairment in 2020 and trends over 30 years, and prevalence of avoidable blindness in relation to VISION 2020: the Right to Sight: an analysis for the Global Burden of Disease Study. Lancet Glob Health. 2021;9:e144–e160. 33275949 10.1016/S2214-109X(20)30489-7 PMC7820391

[30] Ramke J, Evans JR, Habtamu E, Mwangi N, Silva JC, Swenor BK, et al. Grand Challenges in Global Eye Health study group. Grand Challenges in global eye health: a global prioritisation process using Delphi method. Lancet Healthy Longev. 2022;3:e31–e41. 35028632 10.1016/S2666-7568(21)00302-0 PMC8732284

[31] Bourne RRA, Steinmetz, Flaxman J, Briant SR, Taylor HRB PS, Resnikoff S, et al. GBD 2019 Blindness and Vision Impairment Collaborators; Vision Loss Expert Group of the Global Burden of Disease Study. Trends in prevalence of blindness and distance and near vision impairment over 30 years: an analysis for the Global Burden of Disease Study. Lancet Global Health. 2021;9:e130–e143. 33275950 10.1016/S2214-109X(20)30425-3 PMC7820390

[32] International Classification of Diseases 11th edition https://icd.who.int/browse11/l-m/en#/http%253a%252f%252fid.who.int%252ficd%252fentity%252f1103667651 Accessed 14 May 2023.

[33] Vos T, Lim SS, Abbafati C, Abbas KM, Abbasi M, Abbasifard M, et al. GBD 2019 Diseases, Injuries, and Impairments Collaborators. Global burden of 359 diseases, injuries, and impairments, 1990–2019: a systematic analysis for the Global Burden of Disease Study 2019. Lancet. 2020;396:1204–22. 33069326 10.1016/S0140-6736(20)30925-9 PMC7567026

[34] James SL, Abate D, Hassan AK, Abay SM, Abbafati C, et al. GBD 2017 Disease and Injury Incidence and Prevalence Collaborators. Global, regional, and national incidence, prevalence, and years lived with disability for 354 diseases and injuries for 195 countries and territories, 1990–2017: a systematic analysis for the Global Burden of Disease Study 2017. Lancet. 2018;392:1789–858. 30496104 10.1016/S0140-6736(18)32279-7 PMC6227754

[35] Vollset SE, Goren E, Yuan C-W, Cao J, Smith A, Hsiao T, et al. Fertility, mortality, migration, and population scenarios for 195 countries and territories from 2017 to 2100: a forecasting analysis for the Global Burden of Disease Study. Lancet. 2020;396:1285–306. 32679112 10.1016/S0140-6736(20)30677-2 PMC7561721

[36] Stevens GA, Alkema L, Black RE, et al. Guidelines for accurate and transparent health estimates reporting: the GATHER statement. Lancet. 2016;388:e19–23. 27371184 10.1016/S0140-6736(16)30388-9

[37] IAPB Vision Atlas, List of Seven Super Regions https://www.iapb.org/learn/vision-atlas/about/definitions-and-regions/ Accessed 13 June 2022.

[38] Han X, Zhang J, Liu Z, Tan X, Jin G, He M, et al. Real-world visual outcomes of cataract surgery based on population-based studies: a systematic review. Br J Ophthalmol. (2022). 10.1136/bjophthalmol-2021-320997. 10.1136/bjophthalmol-2021-320997 PMC10359559 35410876

[39] United Nations (UN) Department of Economic and Social Affairs World Population Prospects 2022. www.un.org.development.desa.pd/files/wpp2022_summary_of_results.pdf Accessed 13 Feb 2023.

[40] UN World Population Ageing Report 2019 https://www.un.org/en/development/desa/population/publications/pdf/ageing/WorldPopulationAgeing2019-Highlights.pdf Accessed 13 Feb 2023.

[41] Bourne RRA, Cicinelli MV, Sedighi T, Tapply IH, McCormick I, Jonas JB, et al. Effective refractive error coverage in adults aged 50 years and older: estimates from population-based surveys in 61 countries. Lancet Glob Health. 2022;10:e1754–e1763. 36240807 10.1016/S2214-109X(22)00433-8

[42] Morgan IG, French AN, Ashby RS, Guo X, Ding X, He M, et al. The epidemics of myopia: aetiology and prevention. Prog Retin Eye Res. 2018;62:134–49. 28951126 10.1016/j.preteyeres.2017.09.004

[43] Holden BA, Fricke TR, Wilson DA, Jong M, Naidoo KS, Sankaridurg P, et al. Global prevalence of myopia and high myopia and temporal trends from 2000 through 2050. Ophthalmology. 2016;123:1036–42. 26875007 10.1016/j.ophtha.2016.01.006

[44] Courtright P, Lewallen S. Why are we addressing gender issues in vision loss? Community Eye Health. 2009;22:17–9. 19888362 PMC2760274

[45] Fricke TR, Tahhan N, Resnikoff S, Papas E, Burnett A, Ho SM, et al. Global prevalence of presbyopia and vision impairment from uncorrected presbyopia: systematic review, meta-analysis, and modelling. Ophthalmology. 2018;125:1492–9. 29753495 10.1016/j.ophtha.2018.04.013

[46] Ma Q, Chen M, Li D, Zhou R, Du Y, Yin S, et al. Potential productivity loss from uncorrected and under-corrected presbyopia in low- and middle-income countries: a life table modelling study. Front Public Health. 2022;10:983423. 36304252 10.3389/fpubh.2022.983423 PMC9592832

[47] Li M, Xu L, Tan CS, Lanca C, Foo LL, Sabanayagam C, et al. Systematic review and meta-analysis on the impact of COVID-19 pandemic-related lifestyle on myopia. Asia Pac J Ophthalmol. 2022;11:470–80. 10.1097/APO.0000000000000559 36179338

