# Supplementary material for: Global estimates on the number of people blind or visually impaired by Uncorrected Refractive Error: a meta-analysis from 2000 to 2020
Source: Eye (Lond). 2024 Jul 4;38(11):2083–101. doi: 10.1038/s41433-024-03106-0 (PMC11269735; doi:10.1038/s41433-024-03106-0)
Supplement: Supplementary file 1 — Appendix 1 [file 41433_2024_3106_MOESM1_ESM.docx]

**Title: Global estimates on the number of people blind or visually impaired by Uncorrected Refractive Error: A meta-analysis from 2000 to 2020**

**Appendix: Data for 21 GBD World Regions**

**Table 1a:** Number and age-standardised prevalence of people with **blindness (<3/60)** due to URE in 21 GBD Regions in 2020. Data are presented for the whole population and for those 50+ years. Figures in parentheses reflect 95% uncertainty intervals. Count data are presented to three significant figures, and percentages to two decimal places.

|  | Total population 2020 (thousands) | **Number of people with blindness (thousands)** | Number of Men with blindness (thousands) | Number of Women with blindness (thousands) | **Number of people with blindness aged 50+ years (thousands)** | Number of Men with blindness aged 50+ years (thousands) | Number of Women with blindness aged 50+ years (thousands) | **Age standardised prevalence of blindness** | Age standardised prevalence of blindness: Men | Age standardised prevalence of blindness: Women | **Age standardised prevalence of blindness 50+ years** | Age standardised prevalence of blindness in Men 50+ years | Age standardised prevalence of blindness in Women 50+ years | **Percentage of all blindness** |
| --- | --- | --- | --- | --- | --- | --- | --- | --- | --- | --- | --- | --- | --- | --- |
| GLOBAL | 7,890,000 | **3,700**  **(3,100-4,290)** | 1,750  (1,480-2,020) | 1,950  (1,620-2,280) | **2,290**  **(1,790-2,800)** | 1,050  (816-1,270) | 1,250  (975 -1,520) | **0.04%**  **(0.04-0.05)** | 0.04%  (0.04-0.05) | 0.05%  (0.04-0.05) | **0.12%**  **(0.10-0.15)** | 0.12%  (0.09-0.14) | 0.13%  (0.10-0.15) | **8.60%**  **(7.22-9.99)** |
| Andean Latin America | 64,600 | **30.7**  **(24.9-37.0)** | 12.3  (9.87-15.0) | 18.3  (15.0-22.1) | **17.5**  **(13.4-21.8)** | 7.15  (5.40-9.06) | 10.3  (8.01-12.7) | **0.05%**  **(0.04-0.06)** | 0.04%  (0.03-0.05) | 0.06%  (0.05-0.07) | **0.14%**  **(0.11-0.17)** | 0.12%  (0.09-0.15) | 0.16%  (0.12-0.20) | **8.78%**  **(7.13-10.59)** |
| Australasia | 29,000 | **1.70**  **(1.30-2.11)** | 0.766  (0.581-0.958) | 0.934  (0.716-1.16) | **0.946**  **(0.705-1.21)** | 0.428  (0.318-0.554) | 0.518  (0.384-0.658) | **0.00%**  **(0.00-0.01)** | 0.00%  (0.00-0.01) | 0.01%  (0.00-0.01) | **0.01%**  **(0.01-0.01)** | 0.01%  (0.01-0.01) | 0.01%  (0.01-0.01) | **2.47%**  **(1.89-3.06)** |
| Caribbean | 47,200 | **10.2**  **(8.07-12.5)** | 4.85  (3.83-6.01) | 5.36  (4.27-6.54) | **5.36**  **(3.99-6.75)** | 2.48  (1.84-3.16) | 2.88  (2.13-3.62) | **0.02%**  **(0.02-0.02)** | 0.02%  (0.02-0.02) | 0.02%  (0.02-0.02) | **0.05%**  **(0.03-0.06)** | 0.04%  (0.03-0.06) | 0.05%  (0.03-0.06) | **3.93%**  **(3.11-4.80)** |
| Central Asia | 95,000 | **10.0**  **(7.63-12.8)** | 3.94  (2.94-5.11) | 6.09  (4.69-7.63) | **4.75**  **(3.43-6.20)** | 1.65  (1.16-2.22) | 3.10  (2.26-4.03) | **0.01% (0.01-0.01)** | 0.01%  (0.01-0.01) | 0.01%  (0.01-0.02) | **0.03%**  **(0.02-0.03)** | 0.02%  (0.02-0.03) | 0.03%  (0.02-0.04) | **3.33%**  **(2.54-4.24)** |
| Central Europe | 114,000 | **3.81**  **(2.90-4.78)** | 1.57  (1.16-2.03) | 2.24  (1.71-2.80) | **2.30**  **(1.66-3.02)** | 0.821  (0.582-1.10) | 1.48  (1.07-1.92) | **0.00%**  **(0.00-0.00)** | 0.00%  (0.00-0.00) | 0.00%  (0.00-0.00) | **0.01%**  **(0.00-0.01)** | 0.00%  (0.00-0.01) | 0.01%  (0.00-0.01) | **1.16%**  **(0.89-1.46)** |
| Central Latin America | 266,000 | **94.7**  **(78.0-112)** | 40.9  (33.5-48.8) | 53.8  (44.5-63.5) | **54.0**  **(41.8-65.5)** | 22.2  (17.1-27.2) | 31.8  (24.8-38.6) | **0.04% (0.03-0.04)** | 0.03%  (0.03-0.04) | 0.04%  (0.03-0.05) | **0.10%**  **(0.08-0.12)** | 0.09%  (0.07-0.11) | 0.11%  (0.08-0.13) | **7.48%**  **(6.17-8.87)** |
| Central Sub-Saharan Africa | 132,000 | **4.82**  **(3.70-6.13)** | 2.16  (1.64-2.82) | 2.67  (2.06-3.35) | **1.80**  **(1.33-2.31)** | 0.746  (0.544-0.952) | 1.06  (0.780-1.36) | **0.01%**  **(0.00-0.01)** | 0.01%  (0.00-0.01) | 0.01%  (0.01-0.01) | **0.02%**  **(0.01-0.02)** | 0.02%  (0.01-0.02) | 0.02%  (0.01-0.02) | **1.68%**  **(1.29-2.13)** |
| East Asia | 1,500,000 | **1,185**  **(965-1,387)** | 561  (459-656) | 625  (511-732) | **816**  **(639-997)** | 368  (288-450) | 449  (352-546) | **0.06%**  **(0.05-0.07)** | 0.06%  (0.05-0.07) | 0.06%  (0.05-0.07) | **0.16%**  **(0.13-0.20)** | 0.15%  (0.12-0.19) | 0.18%  (0.14-0.21) | **13.05%**  **(10.63-15.26)** |
| Eastern Europe | 209,000 | **15.5**  **(12.5-18.9)** | 8.22  (6.61-10.0) | 7.33  (5.87-8.91) | **9.76**  **(7.42-12.3)** | 4.58  (3.47-5.69) | 5.18  (3.93-6.53) | **0.01%**  **(0.00-0.01)** | 0.01%  (0.01-0.01) | 0.00%  (0.00-0.01) | **0.01%**  **(0.01-0.02)** | 0.02%  (0.01-0.02) | 0.01%  (0.01-0.01) | **1.97%**  **(1.58-2.39)** |
| Eastern Sub-Saharan Africa | 428,000 | **109**  **(89.2-131)** | 54.5  (44.8-65.6) | 54.2  (44.6-65.2) | **37.1**  **28.3-45.5)** | 19.0  (14.4-23.4) | 18.1  (13.8-22.1) | **0.04%**  **(0.03-0.05)** | 0.04%  (0.03-0.05) | 0.04%  (0.03-0.05) | **0.11%**  **(0.08-0.13)** | 0.12%  (0.09-0.14) | 0.10%  (0.08-0.12) | **5.51%**  **(4.53-6.64)** |
| High-income Asia Pacific | 187,000 | **16.1**  **(13.0-19.5)** | 7.25  (5.75-8.84) | 8.82  (7.14-10.6) | **9.39**  **(7.29-11.4)** | 4.23  (3.24-5.25) | 5.15  (4.02-6.25) | **0.01% (0.01-0.01)** | 0.01%  (0.00-0.01) | 0.01%  (0.01-0.01) | **0.01%**  **(0.01-0.01)** | 0.01%  (0.01-0.01) | 0.01%  (0.01-0.01) | **3.00%**  **(2.42-3.64)** |
| High-income North America | 368,000 | **17.0**  **(13.6-21.0)** | 8.58  (6.87-10.6) | 8.45  (6.81-10.4) | **9.05**  **(7.01-11.0)** | 4.52  (3.46-5.57) | 4.53  (3.51-5.57) | **0.00% (0.00-0.01)** | 0.00%  (0.00-0.01) | 0.00%  (0.00-0.01) | **0.01%**  **(0.01-0.01)** | 0.01%  (0.01-0.01) | 0.01%  (0.00-0.01) | **2.39%**  **(1.91-2.95)** |
| North Africa and Middle East | 632,000 | **190**  **(156-229)** | 90.4  (73.6-109) | 99.8  (81.8-120) | **84.2**  **(63.8-103)** | 39.3  (29.6-48.3) | 44.9  (34.1-55.3) | **0.03%**  **(0.03-0.04)** | 0.03%  (0.03-0.04) | 0.04%  (0.03-0.04) | **0.09%**  **(0.07-0.11)** | 0.08%  (0.06-0.10) | 0.09%  (0.07-0.12) | **6.15%**  **(5.03-7.40)** |
| Oceania | 13,500 | **1.03**  **(0.788-1.36)** | 0.497  (0.377-0.648) | 0.537  (0.406-0.706) | **0.302**  **(0.216-0.397)** | 0.150  (0.107-0.197) | 0.153  (0.109-0.200) | **0.01%**  **(0.01-0.01)** | 0.01%  (0.01-0.01) | 0.01%  (0.01-0.01) | **0.02%**  **(0.01-0.02)** | 0.02%  (0.01-0.02) | 0.02%  (0.01-0.03) | **2.63%**  **(2.00-3.47)** |
| South Asia | 1,840,000 | **1,520**  **(1,260-1,770)** | 728  (606-847) | 789  (658-925) | **976**  **(762-1,189)** | 459  (358-558) | 517  (402-633) | **0.10%**  **(0.08-0.12)** | 0.10%  (0.08-0.11) | 0.10%  (0.09-0.12) | **0.33%**  **(0.26-0.40)** | 0.32%  (0.25-0.38) | 0.34%  (0.27-0.42) | **12.71% (10.58-14.82)** |
| Southeast Asia | 679,000 | **221**  **(183-262)** | 96.4  (78.7-115) | 125  (103-148) | **116**  **(89.8-143)** | 47.0  (35.8-58.5) | 69.5  (53.2-85.2) | **0.03%**  **(0.03-0.04)** | 0.03%  (0.02-0.04) | 0.04%  (0.03-0.04) | **0.09%**  **(0.07-0.11)** | 0.07%  (0.06-0.09) | 0.09%  (0.07-0.12) | **3.72%**  **(3.07-4.40)** |
| Southern Latin America | 67,300 | **5.85**  **(4.62-7.19)** | 2.74  (2.17-3.39) | 3.11  (2.46-3.84) | **3.28**  **(2.50-4.06)** | 1.52  (1.16-1.88) | 1.76  (1.32-2.20) | **0.01%**  **(0.01-0.01)** | 0.01%  (0.01-0.01) | 0.01%  (0.01-0.01) | **0.02%**  **(0.01-0.02)** | 0.02% (0.01-0.02) | 0.02% (0.01-0.02) | **3.70%**  **(2.92-4.54)** |
| Southern Sub-Saharan Africa | 81,000 | **15.8**  **(13.0-18.8)** | 6.90  (5.65-8.19) | 8.92  (7.31-10.6) | **9.42**  **(7.22-11.6)** | 3.95  (3.01-4.93) | 5.46  (4.20-6.70) | **0.02%**  **(0.02-0.03)** | 0.02%  (0.02-0.03) | 0.02%  (0.02-0.03) | **0.08%**  **(0.06-0.09)** | 0.08%  (0.06-0.10) | 0.07%  (0.06-0.09) | **3.31%**  **(2.72-3.95)** |
| Tropical Latin America | 224,000 | **82.6**  **(68.4-97.5)** | 38.0  (31.3-45.0) | 44.6  (37.0-52.3) | **49.4**  **(38.3-60.1)** | 21.9  (17.0-26.9) | 27.5  (21.4-33.4) | **0.03%**  **(0.03-0.04)** | 0.03%  (0.03-0.04) | 0.03%  (0.03-0.04) | **0.09%**  **(0.07-0.11)** | 0.09%  (0.07-0.11) | 0.09%  (0.07-0.11) | **4.63%**  **(3.83-5.46)** |
| Western Europe | 437,000 | **39.4**  **(31.2-48.3)** | 17.5  (13.8-21.6) | 21.8  (17.4-26.7) | **23.4**  **(18.0-29.3)** | 10.4  (7.87-13.1) | 13.0  (10.0-16.4) | **0.01%**  **(0.01-0.01)** | 0.01%  (0.00-0.01) | 0.01%  (0.01-0.01) | **0.01%**  **(0.01-0.02)** | 0.01%  (0.01-0.02) | 0.01%  (0.01-0.02) | **2.57%**  **(2.03-3.15)** |
| Western Sub-Saharan Africa | 473,000 | **125**  **(104-150)** | 59.2  (49.3-71.0) | 65.9  (54.6-79.1) | **62.5**  **(47.7-77.6)** | 28.8  (22.0-35.7) | 33.6  (25.5-41.8) | **0.05%**  **(0.04-0.06)** | 0.05%  (0.04-0.06) | 0.05%  (0.04-0.06) | **0.16%**  **(0.12-0.20)** | 0.15%  (0.12-0.19) | 0.16%  (0.12-0.20) | **5.33%**  **(4.42-6.40)** |

**Table 1b:** Number and age-standardised prevalence of people with **MSVI (<6/18 to >/=3/60)** due to URE in 21 GBD Regions in 2020. Data are presented for the whole population and for those 50+ years. Figures in parentheses reflect 95% uncertainty intervals. Count data are presented to three significant figures, and percentages to two decimal places.

|  | Total population 2020 (thousands) | **Number of people with MSVI (thousands)** | Number of Men with MSVI (thousands) | Number of Women with MSVI (thousands) | **Number of people with MSVI aged 50+ years (thousands)** | Number of Men with MSVI aged 50+ years (thousands) | Number of Women with MSVI aged 50+ years (thousands) | **Age standardised prevalence of MSVI** | Age standardised prevalence of MSVI: Men | Age standardised prevalence of MSVI: Women | **Age standardised prevalence of MSVI 50+ years** | Age standardised prevalence of MSVI in Men 50+ years | Age standardised prevalence of MSVI in Women 50+ years | **Percentage of all MSVI** |
| --- | --- | --- | --- | --- | --- | --- | --- | --- | --- | --- | --- | --- | --- | --- |
| GLOBAL | 7,890,000 | **157,000**  **(140,000-176,000)** | 73,300  (65,400-81,900) | 84,100  (74,900-93,900) | **86,100**  **(74,200-101,000)** | 39,000  (33,600-45,810) | 47,100  (40,600-55,200) | **1.91%**  **(1.71-2.13)** | 1.83% (1.63-2.04) | 2.00% (1.78-2.23) | **4.58%**  **(3.96-5.37)** | 4.41%  (3.80-5.16) | 4.75%  (4.09-5.56) | **53.39%**  **(47.56-59.51)** |
| Andean Latin America | 64,600 | **1,610**  **(1,430-1,800)** | 723  (641-808) | 890  (792-992) | **640**  **(548-754)** | 288  (244-341) | 353  (301-415) | **2.58%**  **(2.29-2.88)** | 2.34%  (2.08-2.62) | 2.82%  (2.50-3.14) | **5.06%**  **(4.33-5.96)** | 4.69%  (3.99-5.54) | 5.41%  (4.61-6.35) | **58.51%**  **(51.89-65.21)** |
| Australasia | 29,000 | **434 (386-482)** | 216 (192-242) | 217  (194-242) | **193**  **(164-225)** | 87  (74-102) | 106  (90-125) | **1.38%**  **(1.23-1.55)** | 1.42%  (1.26-1.61) | 1.34%  (1.19-1.50) | **1.75%**  **(1.49-2.05)** | 1.71%  (1.45-2.01) | 1.79%  (1.53-2.10) | **57.89%**  **(51.45-64.25)** |
| Caribbean | 47,200 | **813**  **(714-909)** | 358  (315-402) | 455  (399-509) | **329 (277-390)** | 148  (124-175) | 182  (154-214) | **1.65%**  **(1.45-1.84)** | 1.49%  (1.32-1.67) | 1.80%  (1.59-2.02) | **2.78%**  **(2.33-3.28)** | 2.66%  (2.23-3.15) | 2.89% (2.44-3.41) | **52.34%**  **(45.94-58.50)** |
| Central Asia | 95,000 | **1,550**  **(1,370-1,740)** | 687  (602-773) | 858  (760-973) | **716**  **(593-857)** | 286  (235-345) | 430  (357-517) | **1.73%**  **(1.53-1.94)** | 1.62%  (1.43-1.81) | 1.82%  (1.62-2.05) | **3.99%**  **(3.35-4.73)** | 3.65%  (3.08-4.34) | 4.25%  (3.58-5.05) | **52.37%**  **(46.25-59.11)** |
| Central Europe | 114,000 | **2,120**  **(1,860-2,410)** | 805  (706-913) | 1,320  (1,150-1,500) | **1,540**  **(1,300-1,820)** | 530  (447-636) | 1,010  (853-1,180) | **1.32%**  **(1.17-1.48)** | 1.13%  (1.00-1.28) | 1.48%  (1.31-1.65) | **3.29%**  **(2.80-3.91)** | 2.63%  (2.23-3.13) | 3.82%  (3.25-4.53) | **53.68%**  **(47.05-61.01)** |
| Central Latin America | 266,000 | **5,710**  **(5,030-6,370)** | 2,520  (2,230-2,810) | 3,190  (2,800-3,580) | **2,300**  **(1,970-2,710)** | 1,050  (895-1,240) | 1,260 (1,070-1,480) | **2.16%**  **(1.91-2.41)** | 2.00%  (1.77-2.23) | 2.32%  (2.04-2.60) | **4.19%**  **(3.58-4.92)** | 4.13% (3.54-4.83) | 4.24%  (3.61-4.97) | **57.98%**  **(51.11-64.69)** |
| Central Sub-Saharan Africa | 132,000 | **1,210**  **(1,060-1,360)** | 535  (469-611) | 671  (589-759) | **517**  **(439-620)** | 206  (174-247) | 311  (264-373) | **1.55%**  **(1.37-1.76)** | 1.41%  (1.24-1.59) | 1.67%  (1.47-1.90) | **4.75%**  **(4.07-5.58)** | 4.22%  (3.61-4.93) | 5.18%  (4.42-6.11) | **59.93%**  **(52.58-67.81)** |
| East Asia | 1,500,000 | **27,700**  **(24,500-31,400)** | 12,300  (10,900-14,000) | 15,400  (13,600-17,500) | **20,100**  **(17,200-23,500)** | 8,740  (7,480-10,300) | 11,300  (9,720-13,290) | **1.47%**  **(1.31-1.65)** | 1.34%  (1.19-1.50) | 1.61%  (1.43-1.81) | **4.06%**  **(3.50-4.73)** | 3.66%  (3.15-4.26) | 4.44%  (3.81-5.18) | **51.41%**  **(45.41-58.27)** |
| Eastern Europe | 209,000 | **5,990**  **(5,320-6,780)** | 2,330  (2,060-2,640) | 3,660 (3,250-4,150) | **4,090**  **(3,500-4,810)** | 1,450 (1,230-1,730) | 2,640  (2,250-3,090) | **2.18%**  **(1.94-2.43)** | 2.05%  (1.82-2.28) | 2.29%  (2.04-2.56) | **5.32%**  **(4.57-6.25)** | 4.98%  (4.28-5.88) | 5.59%  (4.80-6.55) | **54.05%**  **(48.00-61.22)** |
| Eastern Sub-Saharan Africa | 428,000 | **3,090**  **(2,710-3,500)** | 1,480  (1,300-1,680) | 1,610  (1,410-1,820) | **874**  **(749-1,020)** | 430  (369-501) | 444  (379-519) | **1.03%**  **(0.91-1.15)** | 1.03%  (0.91-1.14) | 1.03%  (0.92-1.15) | **2.46%**  **(2.13-2.84)** | 2.55%  (2.22-2.94) | 2.38%  (2.05-2.76) | **44.10%**  **(38.61-49.99)** |
| High-income Asia Pacific | 187,000 | **2,750**  **(2,450-3,050)** | 1,340  (1,190-1,480) | 1,410  (1,250-1,580) | **1,670**  **(1,440-1,940)** | 736  (633-854) | 938  (803-1,080) | **1.19%**  **(1.05-1.34)** | 1.25%  (1.10-1.41) | 1.13%  (1.00-1.27) | **1.76%**  **(1.52-2.05)** | 1.75%  (1.51-2.04) | 1.76%  (1.51-2.05) | **51.43%**  **(45.79-57.17)** |
| High-income North America | 368,000 | **4,140**  **(3,650-4,610)** | 1,970  (1,740-2,210) | 2,170 (1,930-2,400) | **1,870**  **(1,610-2,180)** | 782  (671-910) | 1,090 (937-1,270) | **1.04%**  **(0.91-1.18)** | 1.04%  (0.91-1.18) | 1.04%  (0.91-1.17) | **1.34%**  **(1.15-1.56)** | 1.23%  (1.06-1.44) | 1.44%  (1.23-1.68) | **55.65%**  **(49.08-62.01)** |
| North Africa and Middle East | 632,000 | **12,800 (11,400-14,400)** | 6,380 (5,660-7,120) | 6,460  (5,740-7,240) | **4,680**  **(3,960-5,550)** | 2,280 (1,920-2,690) | 2,400  (2,030-2,840) | **2.24%**  **(2.00-2.50)** | 2.17%  (1.93-2.42) | 2.32%  (2.07-2.59) | **4.73%**  **(4.02-5.54)** | 4.57%  (3.88-5.36) | 4.88%  (4.14-5.72) | **58.76%**  **(52.21-65.72)** |
| Oceania | 13,500 | **242**  **(216-272)** | 123  (109-138) | 119  (106-134) | **104**  **(87-124)** | 52  (44-64) | 51  (43-61) | **2.48%**  **(2.20-2.80)** | 2.46%  (2.18-2.76) | 2.51%  (2.23-2.83) | **6.97%**  **(5.91-8.13)** | 6.88%  (5.78-8.11) | 7.06%  (5.99-8.22) | **62.94% (55.98-70.60)** |
| South Asia | 1,840,000 | **53,900 (47,800-60,900)** | 25,600 (22,700-28,900) | 28,200 (25,000-31,900) | **32,100**  **(27,500-37,900)** | 15,600 (13,300-18,4000) | 16,600 (14,200-19,500) | **3.37%**  **(2.99-3.81)** | 3.25%  (2.89-3.65) | 3.51%  (3.11-3.97) | **10.28%**  **(8.82-12.06)** | 10.19%  (8.74-11.89) | 10.40%  (8.93-12.18) | **55.99%**  **(49.64-63.32)** |
| Southeast Asia | 679,000 | **11800**  **(10510-13100)** | 5,730  (5,090-6,390) | 6,050  (5,390-6,730) | **4,850**  **(4,150-5,680)** | 2,320  (1,980-2,720) | 2,540  (2,180-2,950) | **1.75%**  **(1.56-1.95)** | 1.75%  (1.56-1.95) | 1.75%  (1.57-1.96) | **3.40%**  **(2.93-3.93)** | 3.55%  (3.07-4.11) | 3.29%  (2.84-3.80) | **40.95%**  **(36.52-45.65)** |
| Southern Latin America | 67,300 | **1,270**  **(1,120-1,410)** | 599  (530-675) | 666  (591-740) | **508**  **(434-593)** | 199  (169-233) | 309  (265-362) | **1.79%**  **(1.59-2.01)** | 1.78%  (1.57-2.02) | 1.78%  (1.57-1.99) | **2.74%**  **(2.34-3.19)** | 2.43%  (2.07-2.83) | 2.98%  (2.54-3.48) | **59.76%**  **(53.11-66.63)** |
| Southern Sub-Saharan Africa | 81,000 | **978**  **(863-1,100)** | 456  (401-512) | 522  (464-586) | **469**  **(401-555)** | 206  (176-244) | 263  (226-311) | **1.41%**  **(1.25-1.59)** | 1.46%  (1.30-1.64) | 1.38%  (1.23-1.55) | **3.73%**  **(3.21-4.38)** | 4.00%  (3.45-4.66) | 3.57%  (3.07-4.19) | **62.74%**  **(55.42-70.42)** |
| Tropical Latin America | 224,000 | **6,560**  **(5,820-7,310)** | 2,950  (2,620-3,290) | 3,600  (3,200-4,020) | **2,500**  **(2,150-2,930)** | 1,130  (967-1,330) | 1,370  (1,180-1,610) | **2.78%**  **(2.47-3.09)** | 2.60%  (2.32-2.89) | 2.96%  (2.62-3.31) | **4.52%**  **(3.90-5.28)** | 4.51%  (3.88-5.25) | 4.54%  (3.91-5.31) | **63.47%**  **(56.39-70.77)** |
| Western Sub-Saharan Africa | 473,000 | **4,350**  **(3,820-4,920)** | 2,050  (1,800-2,320) | 2,300  (2,020-2,590) | **1,350**  **(1,130-1,600)** | 634  (537-755) | 711  (597-850) | **1.30%**  **(1.14-1.46)** | 1.27%  (1.12-1.43) | 1.32%  (1.16-1.48) | **3.17%**  **(2.69-3.71)** | 3.15%  (2.69-3.71) | 3.18%  (2.69-3.72) | **44.07%**  **(38.72-49.84)** |
| Western Europe | 437,000 | **8,500**  **(7,580-9,430)** | 4,120  (3,660-4,560) | 4,380  (3,910-4,870) | **4,690**  **(4,040-5,430)** | 1,920  (1,650-2,220) | 2,770  (2,380-3,220) | **1.64%**  **(1.45-1.84)** | 1.74%  (1.53-1.96) | 1.53%  (1.36-1.72) | **2.38%**  **(2.05-2.76)** | 2.17%  (1.87-2.53) | 2.55%  (2.19-2.96) | **55.12%**  **(49.14-61.17)** |

**Table 2:** Percentage change in crude prevalence, case number and age-standardised prevalence of MSVI and blindness due to URE in adults aged 50 years and older in the 21 GBD world regions between 2000 and 2020. Percentage change to 1 decimal place and figures in parentheses reflect 95% uncertainty intervals.

|  | **MSVI caused by URE** | | | | | | | | | **Blindness caused by URE** | | | | | | | | |
| --- | --- | --- | --- | --- | --- | --- | --- | --- | --- | --- | --- | --- | --- | --- | --- | --- | --- | --- |
|  | **Percentage change in Crude Prevalence between 2000 and 2020** | | | **Percentage Change in Number of Cases between 2000 and 2020** | | | **Percentage Change in Age standardised prevalence between 2000 and 2020** | | | **Percentage Change in Crude Prevalence between 2000 and 2020** | | | **Percentage Change in Number of Cases between 2000 and 2020** | | | **Percentage Change in Age standardised prevalence between 2000 and 2020** | | |
| Region | Men | Women | **Both** | Men | Women | **Both** | Men | Women | **Both** | Men | Women | **Both** | Men | Women | **Both** | Men | Women | **Both** |
| **GLOBAL** | -2.2% (-2.4 to -2.0) | -2.3%  (-2.5 to-2.1) | **-2.3%**  **(-2.5 to -2.1)** | +72.5% (72.2 to 72.8) | +71.6% (71.3 to 71.9) | **+72.0% (71.7 to 72.3)** | -3.0% (-3.1 to -2.8) | -2.0% (-2.2 to -1.8) | **-2.4% (-2.6 to -2.2)** | -31.0% (-31.2 to -30.8) | -30.6% (-30.8 to -30.5) | **-30.8% (-31.0 to -30.6)** | +21.7% (21.4 to 22.1) | +21.9% (21.6 to 22.2) | **+21.8% (21.5 to 22.2)** | -31.6% (-31.8 to -31.4) | -29.9% (-30.0 to -29.7) | **-30.5% (-30.7 to -30.3)** |
| Andean Latin America | -0.6%  (-0.8 to -0.4) | -3.4%  (-3.5 to -3.2) | -2.1%  (-2.2 to -1.9) | +97.2% (96.8 to 97.6) | +95.0% (94.7 to 95.4) | +96.0% (95.7 to 96.4) | -1.0%  (-1.2 to -0.8) | -3.8%  (-4.0 to -3.6) | -2.5%  (-2.7 to -2.3) | -29.4%  (-29.6 to -29.2) | -32.3%  (-32.5 to -32.2) | -31.1%  (-31.3 to -30.9) | +40.1% (39.7 to 40.5) | +36.6% (36.2 to 36.9) | +38.0% (37.6 to 38.4) | -30.2%  (-30.4 to -30.0) | -33.7%  (-33.9 to -33.5) | -32.2%  (-32.4 to -32.0) |
| Australasia | +8.1% (7.9 to 8.3) | +5.2% (5.0 to 5.4) | +6.5% (6.3 to 6.7) | +77.7% (77.4 to 78.1) | +71.9% (71.5 to 72.2) | +74.5% (74.1 to 74.8) | +1.7% (1.5 to 1.9) | +5.1% (4.9 to 5.3) | +3.4% (3.2 to 3.6) | -4.6%  (-4.9 to -4.3) | -6.4%  (-6.7 to -6.1) | -5.5% (-5.8 to -5.2) | +57.1% (56.5 to 57.6) | +53.0% (52.5 to 53.4) | +54.8% (54.3 to 55.3) | -8.2%  (-8.5 to -7.9) | -7.6%  (-7.9 to -7.3) | -7.7%  (-8.0 to -7.4) |
| Caribbean | -6.0% (-6.2 to -5.8) | -5.9% (-6.1 to -5.7) | -5.9% (-6.1 to -5.7) | +53.2% (52.9 to 53.5) | +57.5% (57.2 to 57.8) | +55.5% (55.2 to 55.8) | -6.0% (-6.2 to -5.8) | -5.9% (-6.1 to -5.7) | -5.9% (-6.1 to -5.7) | -18.6% (-18.8 to -18.3) | -16.8% (-17.1 to -16.6) | -17.6% (-17.9 to -17.4) | +32.8% (32.4 to 33.2) | +39.2% (38.8 to 39.6) | +36.2% (35.8 to 36.6) | -18.6% (-18.8 to -18.3) | -16.9% (-17.1 to -16.6) | -17.7% (-17.9 to -17.4) |
| Central Asia | -7.1% (-7.3 to -6.9) | -8.0% (-8.2 to -7.8) | -7.9% (-8.1 to -7.7) | +70.7% (70.3 to 71.1) | +61.8% (61.5 to 62.2) | +65.2% (64.9 to 65.6) | -3.5% (-3.7 to -3.3) | -4.2% (-4.4 to -4.0) | -4.0% (-4.2 to -3.8) | -17.7% (-18.0 to -17.4) | -17.7% (-18.0 to -17.4) | -18.1% (-18.3 to -17.8) | +51.2% (50.6 to 51.7) | +44.8% (44.3 to 45.3) | +46.9% (46.4 to 47.4) | -15.2% (-15.5 to -14.9) | -12.6% (-12.9 to -12.3) | -13.7% (-14.0 to -13.4) |
| Central Europe | +2.1% (1.9 to 2.3) | +2.7% (2.5 to 2.9) | +2.1 (1.9 to 2.3) | +29.6% (29.4 to 29.9) | +25.7% (25.5 to 26.0) | +27.0% (26.8 to 27.3) | -2.1% (-2.3 to -1.9) | -1.7% (-1.9 to -1.5) | -2.4% (-2.6 to -2.2) | -7.4%  (-7.7 to -7.0) | -5.3%  (-5.6 to -5.0) | -6.5%  (-6.8 to -6.1) | +17.5% (17.1 to 17.9) | +15.9% (15.5 to 16.3) | +16.5% (16.1 to 16.9) | -10.2% (-10.5 to -9.8) | -9.8%  (-10.1 to -9.5) | -10.2% (-10.5 to -9.9) |
| Central Latin America | -9.4% (-9.5 to -9.2) | -8.4% (-8.6 to -8.2) | -8.8% (-9.0 to -8.6) | +87.7% (87.3 to 88.0) | +100.3%  (100.0 to 100.8) | +94.4% (94.0 to 94.8) | -10.5% (-10.7 to -10.4) | -9.8% (-10.0 to -9.6) | -10.1% (-10.3 to -10.0) | -18.7% (-18.9 to -18.5) | -19.9% (-20.1 to -19.7) | -19.2% (-19.4 to -18.9) | +68.4% (67.9 to 68.8) | +75.2% (74.7 to 75.6) | +72.3% (71.9 to 72.8) | -19.9% (-20.1 to -19.7) | -22.3% (-22.5 to -22.1) | -21.0% (-21.2 to -20.8) |
| Central Sub-Saharan Africa | -10.8% (-11.0 to -10.6) | -4.3% (-4.5 to -4.1) | -7.5% (-7.7 to -7.3) | +88.8 (88.4 to 89.2) | +85.2 (84.8 to 85.6) | +86.6% (86.2 to 87.0) | -3.3% (-3.5 to -3.1) | +0.3% (0.1 to 0.5) | -1.4% (-1.6 to -1.2) | -24.2% (-24.4 to -24.0) | -20.1% (-20.3 to -19.8) | -22.1% (-22.4 to -21.9) | +60.4% (59.9 to 61.0) | +54.8% (54.3 to 55.3) | +57.1% (56.5 to 57.6) | -17.5% (-17.7 to -17.2) | -16.5% (-16.8 to -16.3) | -17.1% (-17.3 to -16.8) |
| East Asia | -14.1% (-14.2 to -13.9) | -13.1%  (-13.2 to -12.9) | -13.3% (-13.5 to -13.2) | +69.4% (69.1 to 69.8) | +78.3% (78.0 to 78.6) | +74.3 (74.0 to 74.6) | -16.5% (-16.7 to -16.4) | -13.4% (-13.6 to -13.3) | -14.9% (-15.0 to -14.7) | -40.3%  (-40.4 to -40.1) | -39.0%  (-39.2 to -38.9) | -39.5%  (-39.7 to -39.4) | +17.8% (17.5 to 18.1) | +25.0%  (24.7 to 25.4) | +21.7% (21.3 to 22.0) | -41.4% (-41.6 to -41.3) | -38.9%  (-39.1 to -38.7) | -40.2%  (-40.3 to -40.0) |
| Eastern Europe | +0.3% (0.1 to 0.5) | -1.6% (-1.8 to -1.4) | -1.1% (-1.3 to -0.9) | +21.9% (21.6 to 22.2) | +14.6% (14.4 to 14.8) | +17.1% (16.9 to 17.3) | -1.7%  (-1.9 to -1.5) | -1.5% (-1.7 to -1.3) | -1.8% (-2.0 to -1.6) | -20.5% (-20.7 to -20.3) | -18.4% (-18.7% to -18.2) | -19.1% (-19.4 to -18.9) | -3.4% (-3.7 to -3.1) | -5.0%  (-5.3 to -4.7) | -4.3%  (-4.5 to -4.0) | -21.7%  (-21.9 to -21.5) | -18.9%  (-19.1 to -18.6) | -19.7%  (-20.0 to -19.5) |
| Eastern Sub-Saharan Africa | -9.4% (-9.6 to -9.2) | -9.3% (-9.5 to -9.2) | -9.4% (-9.6 to -9.3) | +65.5% (65.2 to 65.8) | +73.9% (73.6 to 74.2) | +69.7% (69.4 to 70.0) | -8.8%  (-8.9 to -8.6) | -9.2%  (-9.4 to -9.0) | -9.0%  (-9.2 to -8.9) | -16.8% (-17.0 to -16.6) | -20.0%  (-20.2 to -19.8) | -18.5%  (-18.7 to -18.2) | +52.0%  (51.5 to 52.4) | +53.5% (53.1 to 53.9) | +52.7% (52.3 to 53.1) | -15.8% (-16.0 to -15.6) | -20.2% (-20.4 to -19.9) | -18.2% (-18.4 to -18.0) |
| High-Income Asia Pacific | +14.6% (14.4 to 14.8) | +19.2% (19.0 to 19.4) | +17.1% (16.9 to 17.3) | +62.7%  (62.4 to 63.0) | +64.8% (64.5 to 65.1) | +63.9% (63.6 to 64.1) | +1.9% (1.7 to 2.1) | +5.8% (5.6 to 6.0) | +4.0% (3.8 to 4.1) | -10.5%  (-10.7 to -10.2) | +0.6%  (0.4 to 0.9) | -4.7%  (-4.9 to -4.4) | +27.1% (26.8 to 27.5) | +39.1% (38.8 to 39.5) | +33.4% (33.1 to 33.8) | -14.9%  (-15.1 to -14.6) | -3.0% (-3.3 to -2.7) | -8.8%  (-9.1 to -8.5) |
| High-Income North America | +6.9% (6.7 to 7.1) | +3.5% (3.4 to 3.7) | +4.5% (4.3 to 4.7) | +74.4% (74.1 to 74.7) | +57.9%  (57.6 to 58.1) | +64.4% (64.1 to 64.7) | +5.3% (5.1 to 5.5) | +6.0% (5.8 to 6.2) | +5.4 (5.2 to 5.6) | +14.9% (14.6 to 15.3) | +4.4% (4.1 to 4.7) | +9.5% (9.2 to 9.8) | +87.4% (86.9 to 87.9) | +59.2% (58.8 to 59.6) | +72.1% (71.7 to 72.6) | +13.1% (12.8 to 13.4) | +6.2% (5.9 to 6.5) | +10.1% (9.8 to 10.4) |
| North Africa and Middle East | -11.8% (-12.0 to -11.6) | -9.6%  (-9.8 to -9.4) | -10.7% (-10.9 to -10.5) | +83.2% (82.9 to 83.6) | +88.1% (87.8 to 88.5) | +85.7% (85.4% to 86.1) | -9.0%  (-9.2 to -8.8) | -8.1% (-8.3 to -7.9) | -8.5%  (-8.7 to -8.3) | -27.0%  (-27.2 to -26.8) | -20.2%  (-20.5 to -20.0) | -23.5%  (-23.8 to -23.3) | +51.7%  (51.3 to 52.2) | +66.0% (65.5 to 66.4) | +59.0% (58.5 to 59.5) | -23.2%  (-23.4 to -23.0) | -18.3%  (-18.5 to -18.1) | -20.7%  (-20.9 to -20.5) |
| Oceania | -9.3%  (-9.5 to -9.1) | -10.1%  (-10.3 to -10.0) | -9.7%  (-9.9 to -9.5) | +75.1% (74.8 to 75.5) | +75.7% (75.4 to 76.1) | +75.4% (75.1 to 75.8) | -7.2%  (-7.4 to -7.0) | -7.3%  (-7.4 to -7.1) | -7.2% (-7.4 to -7.0) | -17.5% (-17.8 to -17.2) | -15.0% (-15.3 to -14.7) | -16.3% (-16.5 to -16.0) | +59.3% (58.7 to 59.8) | +66.2% (65.6 to 66.7) | +62.7% (62.1 to 63.2) | -16.5%  (-16.8 to -16.2) | -13.2%  (-13.5 to -12.9) | -14.9%  (-15.2 to -14.6) |
| South Asia | -3.3% (-3.5 to -3.2) | -8.5% (-8.7 to -8.3) | -5.9%  (-6.1 to -5.7) | +82.4%  (82.1 to 82.8) | +85.0%  (84.7 to 85.4) | +83.8% (83.4 to 84.1) | -4.3% (-4.5 to -4.1) | -8.9%  (-9.1 to -8.8) | -6.7%  (-6.8 to -6.5) | -39.4%  (-39.5 to -39.2) | -47.2% (-47.4 to -47.1) | -43.6%  (-43.7 to -43.4) | +14.5% (14.2 to 14.8) | +6.7% (6.4 to 7.0) | +10.2 (9.9 to 10.5) | -42.1% (-42.2 to -41.9) | -50.0% (-50.2 to -49.9) | -46.3%  (-46.5 to -46.2) |
| Southeast Asia | -11.4% (-11.6 to -11.3) | -8.5%  (-8.7 to -8.3) | -9.9%  (-10.1 to -9.8) | +77.1%  (76.7 to 77.4) | +83.4%  (83.0 to 83.7) | +80.3%  (80.0 to 80.7) | -10.7%  (-10.8 to -10.5) | -8.1%  (-8.3 to -8.0) | -9.4%  (-9.6 to -9.3) | -30.2%  (-30.4 to -30.0) | -29.7%  (-29.9 to -29.5) | -29.9%  (-30.1 to -29.7) | +39.6%  (39.2 to 39.93) | +40.9%  (40.5 to 41.3) | +40.4% (40.0 to 40.7) | -29.0%  (-29.2 to -28.8) | -30.1%  (-30.3 to -29.9) | -29.5%  (-29.7 to -29.4) |
| Southern Latin America | +0.4%  (0.2 to 0.6) | +1.6  (1.4 to 1.7) | +1.0%  (0.8 to 1.2) | +53.1%  (52.8 to 53.4) | +52.6%  (52.3 to 52.8) | +52.8%  (52.5 to 53.0) | -1.0%  (-1.2 to -0.8) | +0.5% (0.3 to 0.7) | -0.1%  (-0.3 to 0.0) | -7.3%  (-7.5 to -7.0) | -11.6%  (-11.9 to -11.3) | -9.7%  (-9.9 to -9.4) | +41.4% (41.0 to 41.8) | +32.8%  (32.4 to 33.2) | +36.6%  (36.2 to 37.0) | -8.8%  (-9.0 to -8.5) | -12.9%  (-13.2 to -12.6) | -11.0%  (-11.2 to -10.7) |
| Southern Sub-Saharan Africa | -0.5%  (-0.7 to -0.3) | -3.7%  (-3.9 to -3.5) | -2.3% (-2.5to -2.1) | +67.5%  (67.2 to 67.8) | +64.3% (64.0 to 64.6) | +65.7%  (65.3 to 66.0) | -1.0% (-1.2 to -0.8) | -2.9%  (-3.1 to -2.7) | -2.0% (-2.2 to -1.8) | -13.5%  (-13.7 to -13.3) | -21.3%  (-21.5 to -21.1) | -18.2%  (-18.4 to -17.9) | +45.6%  (45.2 to 46.0) | +34.2%  (33.9 to 34.6) | +38.8% (38.4 to 39.2) | -12.2%  (-12.5 to -12.0) | -20.1%  (-20.3 to -19.9) | -16.92 (-17.14to-16.71) |
| Tropical Latin America | +13.6% (13.4 to 13.8) | +15.9%  (15.6 to 16.1) | +14.8% (14.6 to 15.0) | +122.9%  (122.5 to 123.3) | +133.8% (133.4 to 134.2) | +128.7%  (128.3 to 129.2) | +12.1% (11.9 to 12.3) | +14.4%  (14.2 to 14.6) | +13.4% (13.2 to 13.6) | -6.3% (-6.6 to -6.1) | +24.7% (24.4 to 25.0) | +8.6%  (8.3 to 8.9) | +83.8% (83.3 to 84.3) | +151.6% (151.0 to 152.3) | +116.3% (115.7 to 116.9) | -7.3%  (-7.6 to -7.1) | +22.6% (22.3 to 22.9) | +7.2% (6.9 to 7.5) |
| Western Europe | +5.1%  (4.9 to 5.2) | +2.5%  (2.4 to 2.7) | +3.1%  (2.9 to 3.3) | +45.1%  (44.8 to 45.3) | +32.7%  (32.4 to 32.9) | +37.5%  (37.2 to 37.7) | -0.7%  (-0.9 to -0.6) | +0.4%  (0.2 to 0.6) | -0.5%  (-0.7 to -0.4) | -5.2%  (-5.5 to -4.9) | -5.0%  (-5.2 to -4.7) | -5.2%  (-5.5 to -4.9) | +30.9% (30.6 to 31.3) | +23.0%  (22.6 to 23.3) | +26.4%  (26.0 to 26.7) | -8.4%  (-8.7 to -8.2) | -7.0%  (-7.3 to -6.7) | -7.6%  (-7.8 to -7.3) |
| Western Sub-Saharan Africa | -1.5%  (-1.7 to -1.3) | -2.7%  (-2.9 to -2.5) | -1.9%  (-2.1 to -1.7) | +64.7%  (64.3 to 65.0) | +99.5% (99.1 to 99.9) | +81.4%  (81.1 to 81.8) | -0.3%  (-0.5 to -0.1) | -0.6%  (-0.8 to -0.4) | -0.2%  (-0.4 to 0.0) | -6.1%  (-6.3 to -5.8) | -16.0%  (-16.3 to -15.8) | -10.9%  (-11.1 to -10.6) | +57.0% (56.5 to 57.4) | +72.3% (71.8 to 72.8) | +64.9% (64.4 to 65.3) | -4.6%  (-4.9 to -4.3) | -10.4%  (-10.7 to -10.1) | -7.2%  (-7.5 to -7.0) |
