# Supplementary material for: Global estimates on the number of people blind or visually impaired by Uncorrected Refractive Error: a meta-analysis from 2000 to 2020
Source: Eye (Lond). 2024 Jul 4;38(11):2083–101. doi: 10.1038/s41433-024-03106-0 (PMC11269735; doi:10.1038/s41433-024-03106-0)
Supplement: Supplementary file 2 — Appendix 2 - Contributions by Authors [file 41433_2024_3106_MOESM2_ESM.docx]

**Appendix: Contributions by Authors**

# GBD 2019 Blindness and Vision Impairment Collaborators

# Providing data or critical feedback on data sources

Yohannes Habtegiorgis Abate, Mohammad Abdollahi, Ayele Mamo Abebe, Richard Gyan Aboagye, Hiwa Abubaker Ali, Tadele Girum Girum Adal, Nicola J Adderley, Tayo Alex Adekiya, Kishor Adhikari, Qorinah Estiningtyas Sakilah Adnani, Saira Afzal, Bright Opoku Ahinkorah, Ayman Ahmed, Haroon Ahmed, Fares Alahdab, Mohammed Albashtawy, Mohammad T AlBataineh, Tsegaye Alemu, Robert Kaba Alhassan, Syed Shujait Shujait Ali, Louay Almidani, Sofia Androudi, Rodrigo Anguita, Jalal Arabloo, Alessandro Arrigo, Akeza Awealom Asgedom, Tahira Ashraf, Seyyed Shamsadin Athari, Bantalem Tilaye Tilaye Atinafu, Alok Atreya, Ahmed Y Azzam, Sara Bagherieh, Atif Amin Baig, Freddie Bailey, Ovidiu Constantin Baltatu, Shirin Barati, Mainak Bardhan, Till Winfried Bärnighausen, Amadou Barrow, Nebiyou Simegnew Bayileyegn, Akshaya Srikanth Srikanth Bhagavathula, Sonu Bhaskar, Ajay Nagesh Bhat, Gurjit Kaur Bhatti, Mukharram Bikbov, Rupert Bourne, Tasanee Braithwaite, Paul Svitil Briant, Florentino Luciano Caetano dos Santos, Muthia Cenderadewi, Gashaw Sisay Chanie, Dinh-Toi Chu, Maria Vittoria Cicinelli, Nathan G Congdon, Natália Cruz-Martins, Xiaochen Dai, Lalit Dandona, Rakhi Dandona, Nikolaos Dervenis, Thanh Chi Do, Thao Huynh Phuong Do, Joshua R Ehrlich, Michael Ekholuenetale, Mohammad Hassan Emamian, Adeniyi Francis Fagbamigbe, Hossein Farrokhpour, Ali Fatehizadeh, Alireza Feizkhah, Arthur G Fernandes, Lorenzo Ferro Desideri, João M. Furtado, Muktar A Gadanya, Tilaye Gebru Gebi, Bardiya Ghaderi Yazdi, Sherief Ghozy, Mahaveer Golechha, Pouya Goleij, Sapna Gupta, Vivek Kumar Gupta, Teklehaimanot Gereziher Haile, Ahmed I Hasaballah, Demisu Zenbaba Heyi, Mehdi Hosseinzadeh, Hong-Han Huynh, Sheikh Mohammed Shariful Islam, Mihajlo Jakovljevic, Sathish Kumar Jayapal, Shubha Jayaram, Jost B Jonas, Charity Ehimwenma Joshua, Sagarika Kamath, Himal Kandel, Ibraheem M Karaye, Soujanya Kaup, Harkiran Kaur, Gbenga A. Kayode, John H Kempen, Yousef Saleh Khader, Himanshu Khajuria, Rovshan Khalilov, Ajmal Khan, Mahalaqua Nazli Khatib, Yun Jin Kim, Adnan Kisa, Sezer Kisa, Soewarta Kosen, Kewal Krishan, Burcu Kucuk Bicer, L V Simhachalam Kutikuppala, Chandrakant Lahariya, Tri Laksono, Dharmesh Kumar Lal, Van Charles Lansingh, Janet L. Leasher, Munjae Lee, Seung Won Lee, Stephen S Lim, Julie-Anne Little, Xuefeng Liu, Sandeep B Maharaj, Kashish Malhotra, Tauqeer Hussain Mallhi, Roy Rillera Marzo, Andrea Maugeri, Awoke Misganaw, Soheil Mohammadi, Abdollah Mohammadian-Hafshejani, Hoda Mojiri-forushani, Ali H Mokdad, Maryam Moradi, Christopher J L Murray, Kovin S Naidoo, Ganesh R Naik, Zuhair S Natto, Muhammad Naveed, Biswa Prakash Nayak, Dang H Nguyen, Hien Quang Nguyen, Phat Tuan Nguyen, Van Thanh Nguyen, Robina Khan Niazi, Bogdan Oancea, Osaretin Christabel Okonji, Andrew T Olagunju, Obinna E Onwujekwe, Uchechukwu Levi Osuagwu, Mayowa O Owolabi, Jagadish Rao Padubidri, Songhomitra Panda-Jonas, Anamika Pandey, Shahina Pardhan, Amirhossein Parsaei, Jay Patel, Shrikant Pawar, Arokiasamy Perianayagam, Konrad Pesudovs, Hoang Tran Pham, Shakthi Kumaran Ramasamy, Elrashdy Moustafa Mohamed Redwan, Serge Resnikoff, Jefferson Antonio Buendia Rodriguez, Zahra Saadatian, Siamak Sabour, Basema Saddik, Umar Saeed, Narjes Saheb Sharif-Askari, Mohammad Ali Sahraian, Sara Samadzadeh, Abdallah M Samy, Mete Saylan, Tabassom Sedighi, Yashendra Sethi, Allen Seylani, Masood Ali Shaikh, Aminu Shittu, Parnian Shobeiri, Juan Carlos Silva, Jasvinder A. Singh, Paramdeep Singh, Houman Sotoudeh, Chandrashekhar T Sreeramareddy, Jaimie D Steinmetz, Nina Tahhan, Ian Tapply, Hugh R Taylor, Tala Tillawi, Aristidis Tsatsakis, Guesh Mebrahtom Tsegay, Sree Sudha Ty, Muhammad Umair, Sahel Valadan Tahbaz, Maria Viskadourou, Theo Vos, Gizachew Tadesse Wassie, Guadie Sharew Wondimagegn, Galal Yahya, Naohiro Yonemoto, Mikhail Sergeevich Zastrozhin, Zhi-Jiang Zhang, Magdalena Zielińska, and Mohammad Zoladl.

# Developing methods or computational machinery

Zahra Abbasi Dolatabadi, Ayele Mamo Abebe, Hiwa Abubaker Ali, Qorinah Estiningtyas Sakilah Adnani, Mohammed Albashtawy, Tsegaye Alemu, Aleksandr Y Aravkin, Alessandro Arrigo, Ahmed Y Azzam, Shirin Barati, Abhishek Bhadra, Akshaya Srikanth Srikanth Bhagavathula, Rupert Bourne, Paul Svitil Briant, Gashaw Sisay Chanie, Kaleb Coberly, Xiaochen Dai, Thanh Chi Do, Mehdi Emamverdi, Ali Fatehizadeh, Alireza Feizkhah, Tilaye Gebru Gebi, Sherief Ghozy, Ali Golchin, Teklehaimanot Gereziher Haile, Mehdi Hosseinzadeh, Hong-Han Huynh, Sathish Kumar Jayapal, Jost B Jonas, Rovshan Khalilov, Mahalaqua Nazli Khatib, Adnan Kisa, Chandrakant Lahariya, Hoda Mojiri-forushani, Ali H Mokdad, Hamed Momeni-Moghaddam, Christopher J L Murray, Kovin S Naidoo, Dang H Nguyen, Hien Quang Nguyen, Phat Tuan Nguyen, Van Thanh Nguyen, Michal Ordak, Songhomitra Panda-Jonas, Amirhossein Parsaei, Hoang Tran Pham, Jefferson Antonio Buendia Rodriguez, Umar Saeed, Abdallah M Samy, Parnian Shobeiri, Jaimie D Steinmetz, Ian Tapply, Tala Tillawi, Muhammad Umair, and Theo Vos.

# Providing critical feedback on methods or results

Yohannes Habtegiorgis Abate, Zahra Abbasi Dolatabadi, Michael Abdelmasseh, Mohammad Abdollahi, Ayele Mamo Abebe, Olumide Abiodun, Richard Gyan Aboagye, Woldu Aberhe Abrha, Hiwa Abubaker Ali, Eman Abu-Gharbieh, Salahdein Aburuz, Tadele Girum Girum Adal, Lawan Hassan Adamu, Nicola J Adderley, Isaac Yeboah Addo, Kishor Adhikari, Qorinah Estiningtyas Sakilah Adnani, Saira Afzal, Shahin Aghamiri, Antonella Agodi, Williams Agyemang-Duah, Bright Opoku Ahinkorah, Aqeel Ahmad, Hooman Ahmadzadeh, Ayman Ahmed, Haroon Ahmed, Fares Alahdab, Mohammed Albashtawy, Mohammad T AlBataineh, Tsegaye Alemu, Ahmad Samir Alfaar, Robert Kaba Alhassan, Abid Ali, Syed Shujait Shujait Ali, Louay Almidani, Karem H Alzoubi, Sofia Androudi, Rodrigo Anguita, Anayochukwu Edward Anyasodor, Jalal Arabloo, Damelash Areda, Alessandro Arrigo, Akeza Awealom Asgedom, Mubarek Yesse Ashemo, Tahira Ashraf, Seyyed Shamsadin Athari, Bantalem Tilaye Tilaye Atinafu, Maha Moh'd Wahbi Atout, Alok Atreya, Haleh Ayatollahi, Ahmed Y Azzam, Sara Bagherieh, Ruhai Bai, Atif Amin Baig, Freddie Bailey, Ovidiu Constantin Baltatu, Shirin Barati, Martina Barchitta, Mainak Bardhan, Till Winfried Bärnighausen, Amadou Barrow, Nebiyou Simegnew Bayileyegn, Alemshet Yirga Berhie, Abhishek Bhadra, Akshaya Srikanth Srikanth Bhagavathula, Pankaj Bhardwaj, Sonu Bhaskar, Ajay Nagesh Bhat, Gurjit Kaur Bhatti, Mukharram Bikbov, Marina G Birck, Rupert Bourne, Tasanee Braithwaite, Paul Svitil Briant, Yasser Bustanji, Zahid A Butt, Florentino Luciano Caetano dos Santos, Muthia Cenderadewi, Gashaw Sisay Chanie, Nicolas Cherbuin, Dinh-Toi Chu, Nathan G Congdon, Natália Cruz-Martins, Omid Dadras, Xiaochen Dai, Lalit Dandona, Rakhi Dandona, Ana Maria Dascalu, Tadesse Asmamaw Dejenie, Dessalegn Demeke, Nikolaos Dervenis, Vinoth Gnana Chellaiyan Devanbu, Daniel Diaz, Mengistie Diress, Thanh Chi Do, Thao Huynh Phuong Do, Arkadiusz Marian Dziedzic, Hisham Atan Edinur, Michael Ekholuenetale, Hala Rashad Elhabashy, Muhammed Elhadi, Mohammad Hassan Emamian, Mehdi Emamverdi, Adeniyi Francis Fagbamigbe, Hossein Farrokhpour, Ali Fatehizadeh, Lorenzo Ferro Desideri, Getahun Fetensa, Florian Fischer, João M. Furtado, Muktar A Gadanya, Abhay Motiramji Gaidhane, Tilaye Gebru Gebi, Mesfin Gebrehiwot, Bardiya Ghaderi Yazdi, Khalil Ghasemi Falavarjani, Fariba Ghassemi, Sherief Ghozy, Ali Golchin, Mahaveer Golechha, Shi-Yang Guan, Sapna Gupta, Vivek Kumar Gupta, Rasool Haddadi, Teklehaimanot Gereziher Haile, Mehdi Harorani, Ahmed I Hasaballah, Ikramul Hasan, Hamidreza Hasani, Hossein Hassanian-Moghaddam, Golnaz Heidari, Demisu Zenbaba Heyi, Ramesh Holla, Mehdi Hosseinzadeh, Chengxi Hu, Hong-Han Huynh, Bing-Fang Hwang, Irena M. Ilic, Mustapha Immurana, Sheikh Mohammed Shariful Islam, Louis Jacob, Mihajlo Jakovljevic, Sathish Kumar Jayapal, Shubha Jayaram, Jost B Jonas, Nitin Joseph, Charity Ehimwenma Joshua, Sagarika Kamath, Himal Kandel, Ibraheem M Karaye, Soujanya Kaup, Harkiran Kaur, Navjot Kaur, Gbenga A. Kayode, John H Kempen, Himanshu Khajuria, Rovshan Khalilov, Ajmal Khan, Moawiah Mohammad Khatatbeh, Mahalaqua Nazli Khatib, Biruk Getahun Kibret, Yun Jin Kim, Adnan Kisa, Sezer Kisa, Ai Koyanagi, Kewal Krishan, Nithin Kumar, L V Simhachalam Kutikuppala, Chandrakant Lahariya, Tri Laksono, Dharmesh Kumar Lal, Van Charles Lansingh, Janet L. Leasher, Seung Won Lee, Wei-Chen Lee, Stephen S Lim, Julie-Anne Little, Xuefeng Liu, Alireza Mahmoudi, Kashish Malhotra, Ahmad Azam Malik, Iram Malik, Tauqeer Hussain Mallhi, Vahid Mansouri, Roy Rillera Marzo, Andrea Maugeri, Gebrekiros Gebremichael Meles, Abera M Mersha, Tomislav Mestrovic, Ted R Miller, Mehdi Mirzaei, Awoke Misganaw, Sanjeev Misra, Prasanna Mithra, Soheil Mohammadi, Abdollah Mohammadian-Hafshejani, Maryam Mohammadzadeh, Ali H Mokdad, Hamed Momeni-Moghaddam, Fateme Montazeri, Maryam Moradi, Christopher J L Murray, Kovin S Naidoo, Ganesh R Naik, Gurudatta Naik, Zuhair S Natto, Muhammad Naveed, Biswa Prakash Nayak, Hadush Negash, Seyed Aria Nejadghaderi, Dang H Nguyen, Duc Hoang Nguyen, Hien Quang Nguyen, Phat Tuan Nguyen, Van Thanh Nguyen, Robina Khan Niazi, Efaq Ali Noman, Bogdan Oancea, Osaretin Christabel Okonji, Andrew T Olagunju, Isaac Iyinoluwa Olufadewa, Obinna E Onwujekwe, Abdulahi Opejin Opejin, Michal Ordak, Uchechukwu Levi Osuagwu, Nikita Otstavnov, Mayowa O Owolabi, Jagadish Rao Padubidri, Songhomitra Panda-Jonas, Anamika Pandey, Shahina Pardhan, Amirhossein Parsaei, Jay Patel, Shrikant Pawar, Arokiasamy Perianayagam, Navaraj Perumalsamy, Konrad Pesudovs, Ionela-Roxana Petcu, Hoang Tran Pham, Mohsen Pourazizi, Elton Junio Sady Prates, Ibrahim Qattea, Pankaja Raghav Raghav, Mosiur Rahman, Shakthi Kumaran Ramasamy, Premkumar Ramasubramani, Mohammad-Mahdi Rashidi, Elrashdy Moustafa Mohamed Redwan, Nazila Rezaei, Zahra Saadatian, Siamak Sabour, Basema Saddik, Umar Saeed, Sare Safi, Fatemeh Saheb Sharif-Askari, Narjes Saheb Sharif-Askari, Mohammad Ali Sahraian, Joseph W Sakshaug, Mohamed A. Saleh, Sara Samadzadeh, Yoseph Leonardo Samodra, Abdallah M Samy, Mete Saylan, Yashendra Sethi, Moyad jamal Shahwan, Masood Ali Shaikh, Muhammad Aaqib Shamim, Bereket Beyene Shashamo, Wondimeneh Shibabaw Shiferaw, Mika Shigematsu, Aminu Shittu, Seyed Afshin Shorofi, Migbar Mekonnen Sibhat, Emmanuel Edwar Siddig, Jasvinder A. Singh, Paramdeep Singh, Houman Sotoudeh, Chandrashekhar T Sreeramareddy, Jaimie D Steinmetz, Mohammad Tabish, Majid Taheri, Yao Tan, Ian Tapply, Birhan Tsegaw Taye, Mohamad-Hani Temsah, Jansje Henny Vera Ticoalu, Tala Tillawi, Misganaw Guadie Tiruneh, Guesh Mebrahtom Tsegay, Miltiadis K Tsilimbaris, Chukwudi S Ubah, Muhammad Umair, Sahel Valadan Tahbaz, Rohollah Valizadeh, Maria Viskadourou, Theo Vos, Gizachew Tadesse Wassie, Nuwan Darshana Wickramasinghe, Guadie Sharew Wondimagegn, Galal Yahya, Yao Yao, Arzu Yiğit, Yazachew Yismaw, Naohiro Yonemoto, Yuyi You, Mikhail Sergeevich Zastrozhin, Getachew Assefa Zenebe, Zhi-Jiang Zhang, Hanqing Zhao, Magdalena Zielińska, and Mohammad Zoladl.

# Drafting the work or revising it critically for important intellectual content

Yohannes Habtegiorgis Abate, Michael Abdelmasseh, Ayele Mamo Abebe, Olumide Abiodun, Eman Abu-Gharbieh, Salahdein Aburuz, Lawan Hassan Adamu, Nicola J Adderley, Isaac Yeboah Addo, Kishor Adhikari, Qorinah Estiningtyas Sakilah Adnani, Saira Afzal, Antonella Agodi, Bright Opoku Ahinkorah, Hooman Ahmadzadeh, Ayman Ahmed, Haroon Ahmed, Fares Alahdab, Mohammed Albashtawy, Mohammad T AlBataineh, Tsegaye Alemu, Ahmad Samir Alfaar, Fadwa Alhalaiqa Naji Alhalaiqa, Robert Kaba Alhassan, Abid Ali, Karem H Alzoubi, Rodrigo Anguita, Abhishek Anil, Anayochukwu Edward Anyasodor, Jalal Arabloo, Alessandro Arrigo, Akeza Awealom Asgedom, Seyyed Shamsadin Athari, Bantalem Tilaye Tilaye Atinafu, Maha Moh'd Wahbi Atout, Alok Atreya, Ahmed Y Azzam, Sara Bagherieh, Ruhai Bai, Atif Amin Baig, Freddie Bailey, Ovidiu Constantin Baltatu, Shirin Barati, Martina Barchitta, Mainak Bardhan, Till Winfried Bärnighausen, Amadou Barrow, Maurizio Battaglia Parodi, Abhishek Bhadra, Akshaya Srikanth Srikanth Bhagavathula, Sonu Bhaskar, Ajay Nagesh Bhat, Gurjit Kaur Bhatti, Mukharram Bikbov, Marina G Birck, Rupert Bourne, Tasanee Braithwaite, Yasser Bustanji, Florentino Luciano Caetano dos Santos, Vera L A Carneiro, Muthia Cenderadewi, Gashaw Sisay Chanie, Nicolas Cherbuin, Dinh-Toi Chu, Nathan G Congdon, Natália Cruz-Martins, Ana Maria Dascalu, Anna Dastiridou, Tadesse Asmamaw Dejenie, Diriba Dereje, Nikolaos Dervenis, Vinoth Gnana Chellaiyan Devanbu, Daniel Diaz, Thanh Chi Do, Arkadiusz Marian Dziedzic, Joshua R Ehrlich, Muhammed Elhadi, Mohammad Hassan Emamian, Mehdi Emamverdi, Azin Etemadimanesh, Adeniyi Francis Fagbamigbe, Ali Fatehizadeh, Arthur G Fernandes, Lorenzo Ferro Desideri, Getahun Fetensa, Florian Fischer, Ali Forouhari, João M. Furtado, Muktar A Gadanya, Aravind P Gandhi, Tilaye Gebru Gebi, Mesfin Gebrehiwot, Gebreamlak Gebremedhn Gebremeskel, Yibeltal Yismaw Gela, Bardiya Ghaderi Yazdi, Khalil Ghasemi Falavarjani, Fariba Ghassemi, Sherief Ghozy, Ali Golchin, Shi-Yang Guan, Sapna Gupta, Vivek Kumar Gupta, Teklehaimanot Gereziher Haile, Billy Randall Hammond, Ahmed I Hasaballah, Ikramul Hasan, Hamidreza Hasani, Golnaz Heidari, Demisu Zenbaba Heyi, Ramesh Holla, Hong-Han Huynh, Ivo Iavicoli, Irena M. Ilic, Mustapha Immurana, Sheikh Mohammed Shariful Islam, Louis Jacob, Abdollah Jafarzadeh, Mihajlo Jakovljevic, Manthan Dilipkumar Janodia, Sathish Kumar Jayapal, Shubha Jayaram, Jost B Jonas, Nitin Joseph, Sagarika Kamath, Himal Kandel, Hengameh Kasraei, Soujanya Kaup, Navjot Kaur, Gbenga A. Kayode, John H Kempen, Yousef Saleh Khader, Himanshu Khajuria, Rovshan Khalilov, Ajmal Khan, Moawiah Mohammad Khatatbeh, Mahalaqua Nazli Khatib, Adnan Kisa, Sezer Kisa, Ai Koyanagi, Kewal Krishan, Burcu Kucuk Bicer, L V Simhachalam Kutikuppala, Chandrakant Lahariya, Janet L. Leasher, Julie-Anne Little, Xuefeng Liu, Ahmad Azam Malik, Tauqeer Hussain Mallhi, Vahid Mansouri, Roy Rillera Marzo, Andrea Maugeri, Gebrekiros Gebremichael Meles, Tomislav Mestrovic, Ted R Miller, Mehdi Mirzaei, Awoke Misganaw, Prasanna Mithra, Soheil Mohammadi, Abdollah Mohammadian-Hafshejani, Maryam Mohammadzadeh, Ali H Mokdad, Hamed Momeni-Moghaddam, Fateme Montazeri, Maryam Moradi, Parsa Mousavi, Christopher J L Murray, Kovin S Naidoo, Zuhair S Natto, Biswa Prakash Nayak, Hadush Negash, Seyed Aria Nejadghaderi, Dang H Nguyen, Duc Hoang Nguyen, Hien Quang Nguyen, Phat Tuan Nguyen, Van Thanh Nguyen, Robina Khan Niazi, Bogdan Oancea, Osaretin Christabel Okonji, Andrew T Olagunju, Obinna E Onwujekwe, Abdulahi Opejin Opejin, Michal Ordak, Uchechukwu Levi Osuagwu, Nikita Otstavnov, Mayowa O Owolabi, Jagadish Rao Padubidri, Songhomitra Panda-Jonas, Shahina Pardhan, Amirhossein Parsaei, Jay Patel, Shrikant Pawar, Konrad Pesudovs, Ionela-Roxana Petcu, Hoang Tran Pham, Mohsen Pourazizi, Elton Junio Sady Prates, Ibrahim Qattea, Pankaja Raghav Raghav, Mohammad Hifz Ur Rahman, Premkumar Ramasubramani, Elrashdy Moustafa Mohamed Redwan, Serge Resnikoff, Nazila Rezaei, Jefferson Antonio Buendia Rodriguez, Zahra Saadatian, Basema Saddik, Umar Saeed, Sare Safi, Amene Saghazadeh, Fatemeh Saheb Sharif-Askari, Narjes Saheb Sharif-Askari, Amirhossein Sahebkar, Mohammad Ali Sahraian, Sara Samadzadeh, Yoseph Leonardo Samodra, Abdallah M Samy, Mete Saylan, Siddharthan Selvaraj, Yashendra Sethi, Allen Seylani, Moyad jamal Shahwan, Muhammad Aaqib Shamim, Bereket Beyene Shashamo, Mika Shigematsu, Aminu Shittu, Parnian Shobeiri, Seyed Afshin Shorofi, Migbar Mekonnen Sibhat, Emmanuel Edwar Siddig, Jasvinder A. Singh, Paramdeep Singh, Raúl A R C Sousa, Chandrashekhar T Sreeramareddy, Mohammad Tabish, Majid Taheri, Yao Tan, Hugh R Taylor, Mohamad-Hani Temsah, Jansje Henny Vera Ticoalu, Tala Tillawi, Aristidis Tsatsakis, Sree Sudha Ty, Chukwudi S Ubah, Muhammad Umair, Sahel Valadan Tahbaz, Theo Vos, Nuwan Darshana Wickramasinghe, Guadie Sharew Wondimagegn, Galal Yahya, Lin Yang, Yao Yao, Arzu Yiğit, Naohiro Yonemoto, Mikhail Sergeevich Zastrozhin, Getachew Assefa Zenebe, Zhi-Jiang Zhang, Hanqing Zhao, Magdalena Zielińska, and Mohammad Zoladl.

# Managing the estimation or publications process

Saira Afzal, Mohammed Albashtawy, Alessandro Arrigo, Ahmed Y Azzam, Shirin Barati, Rupert Bourne, Nathan G Congdon, Thanh Chi Do, Ali Fatehizadeh, Teklehaimanot Gereziher Haile, Hong-Han Huynh, Sathish Kumar Jayapal, Jost B Jonas, Soujanya Kaup, Mahalaqua Nazli Khatib, Chandrakant Lahariya, Julie-Anne Little, Ali H Mokdad, Maryam Moradi, Christopher J L Murray, Kovin S Naidoo, Hien Quang Nguyen, Phat Tuan Nguyen, Van Thanh Nguyen, Hoang Tran Pham, Jefferson Antonio Buendia Rodriguez, Sara Samadzadeh, Abdallah M Samy, Muhammad Umair, Theo Vos, and Mikhail Sergeevich Zastrozhin.

Vision Loss Expert Group of the Global Burden of Disease Study

### Providing data or critical feedback on data sources

Alessandro Arrigo, Maurizio Battaglia Parodi, Mukharram M Bikbov, Rupert R A Bourne, Tasanee Braithwaite, Alain Bron, Ching-Yu Cheng, Maria Vittoria Cicinelli, Nathan Congdon, Monte A Del Monte, Joshua R Ehrlich, Leon B Ellwein, Arthur Fernandes, Seth Flaxman, Tim Fricke, David Friedman, João M Furtado, Gus Gazzard, Ronnie George, M Elizabeth Hartnett, Jost B Jonas, Rim Kahloun, John H Kempen, Moncef Khairallah, Rohit C Khanna, Judy E Kim, Van Charles Lansingh, Janet Leasher, Nicolas Leveziel, Julie-Anne Little, Kovin S Naidoo, Vinay Nangia, Michal Nowak, Konrad Pesudovs, Tunde Peto, Pradeep Ramulu, Serge Resnikoff, Tabassom Sedighi, Ian Tapply, Hugh Taylor, Fotis Topouzis, Miltiadis Tsilimbaris, Ya Xing Wang, Ningli Wang

### Developing methods or computational machinery

Rupert R A Bourne, Jost B Jonas, Ian Tapply

### Providing critical feedback on methods or results

Alessandro Arrigo, Mukharram M Bikbov, Rupert R A Bourne, Tasanee Braithwaite, Monte A Del Monte, David Friedman, João M Furtado, M Elizabeth Hartnett, Jost B Jonas, Rim Kahloun, John H Kempen, Janet Leasher, Julie-Anne Little, Kovin S Naidoo, Konrad Pesudovs, Serge Resnikoff, Ian Tapply, Ningli Wang

### Drafting the work or revising it critically for important intellectual content

Alessandro Arrigo, Mukharram M Bikbov, Rupert R A Bourne, Tasanee Braithwaite, Nathan Congdon, Monte A Del Monte, Ronnie George, M Elizabeth Hartnett, Jost B Jonas, Janet Leasher, Julie-Anne Little, Konrad Pesudovs

### Managing the estimation or publications process

Rupert R A Bourne, Jost B Jonas, Julie-Anne Little
